# Response of Chironomidae (Diptera) to DDT, Mercury, and Arsenic Legacy Pollution in Sediments of the Toce River (Northern Italy)

**DOI:** 10.3390/insects15030148

**Published:** 2024-02-22

**Authors:** Laura Marziali, Niccolò Pirola, Alfredo Schiavon, Bruno Rossaro

**Affiliations:** 1 National Research Council-Water Research Institute (CNR-IRSA), Via del Mulino 19, 20861 Brugherio, MB, Italy; niccolo.pirola94@gmail.com (N.P.); alfredo.schiavon@igb-berlin.de (A.S.); 2 Department of Biology, Chemistry, and Pharmacy, Freie Universität Berlin, Arnimallee 22, 14195 Berlin, Germany; 3 Department of Agricultural and Environmental Sciences (DISAA), University of Milan, Via Celoria 2, 20133 Milan, MI, Italy; bruno.rossaro@unimi.it

**Keywords:** chironomid taxa assemblages, macroinvertebrate communities, sediment contamination, bioindicators, multivariate analysis, Self-Organizing Maps

## Abstract

**Simple Summary:**

Historical contamination may still pose a toxicity risk to living organisms. The insecticide DDT and the heavy metals mercury (Hg) and arsenic (As) were discharged in the last century (ca. 1915–1996) into the Toce River (Northern Italy) by a factory, and nowadays residual contamination is still detectable in bottom sediments. Various aquatic invertebrates, including non-biting midges (Diptera, Chironomidae), inhabit these sediments and can serve as bioindicators of potential adverse effects caused by the toxicants. We collected and analyzed sediments and Chironomidae at riverine sites located upstream and downstream of the industrial area. Contamination downstream reaches levels potentially toxic for aquatic organisms. A total of 32 chironomid species were identified. Most species were distributed among different sites based on natural factors such as water temperature, water flow, and substrate type. Few species proved to be sensitive to contamination, such as *Diamesa* spp., *Sympotthastia spinifera*, and *Prodiamesa olivacea* to DDT; *Potthastia longimanus* to Hg; *Odontomesa fulva* and *Microtendipes pedellus* to As. These species may serve as bioindicators of contamination in other freshwater ecosystems.

**Abstract:**

The Toce River (Northern Italy) is characterized by legacy contamination of dichloro-diphenyl-trichloroethane (DDT), mercury, and arsenic deriving from an industrial plant active between ca. 1915 and 1996. Chironomidae taxa assemblages and sediments were collected in 2014 and 2019 upstream and downstream of the industrial area to analyze species responses to toxic substances in a river stretch with relatively uniform natural (i.e., hydro-morphological) characteristics. A total of 32 chironomid taxa were identified. Sediment concentrations reached levels potentially toxic for benthic invertebrates: 15.7 µg kg^−1^ 1% organic carbon for DDT, 197 µg kg^−1^ dry weight (d.w.) for Hg, and 55.7 mg kg^−1^ d.w. for As. Canonical Correspondence Analysis (CCA) revealed a predominant seasonal gradient, followed by an upstream-downstream gradient. Partial CCA indicated that 5.2% of the total variation was associated with sediment contamination. Self-Organizing Maps (SOMs) were used to represent species responses to toxicants. Most species appeared to be tolerant, e.g., *Chironomus riparius*, *Micropsectra atrofasciata*, *Conchapelopia pallidula*, and *Polypedilum* spp. Sensitivity to contaminants was observed in only a few species: *Diamesa* spp., *Sympotthastia spinifera,* and *Prodiamesa olivacea* to DDT; *Potthastia longimanus* to Hg; *Odontomesa fulva* and *Microtendipes pedellus* to As. The chironomid community was characterized in presence of contamination levels commonly observed in freshwater ecosystems.

## 1. Introduction

The response of chironomids from different lentic and lotic habitats to environmental factors has been extensively analyzed in previous studies [1]. Different species have been shown to be sensitive to natural factors, such as water temperature, oxygen level, river flow, substrate particle size, and nutrient content [2]. Thus, the analysis of chironomid taxa assemblages may reveal anthropogenic alterations of these parameters. For example, the relationship with water quality, organic pollution, nutrient enrichment, and oxygen shortage has been observed [3,4,5].

While these organisms are good indicators of overall anthropogenic pressure, their relationship to specific stressors has often proven difficult to disentangle. Regarding the effects of toxic substances, such as trace metals, pesticides, or other pollutants, responses have primarily been described through analysis of specific biomarkers, mouth part deformities [6,7], gene expression [8,9], or alterations in life history parameters [10] in laboratory [11] or in microcosm studies [12]. Conversely, field studies at the community level have rarely shown clear relationships between species and toxicants, as other variables may act as confounding factors. For example, long-term shifts in taxa assemblages were described along sediment cores collected from historically contaminated sites, yet variations in contaminant concentrations have often been associated with trends of other significant drivers, such as nutrient loads or water temperature [13,14]. Studies conducted in contaminated rivers have frequently revealed community structures strongly correlated with variables expressing the longitudinal axis of the river, such as distance from the source, altitude, and water temperature [15]. However, chironomids demonstrate high potential as bioindicators of the presence of toxicants, being one of the most abundant and species-rich macroinvertebrate groups at sites with elevated levels of contamination [16].

The aim of this study is to find potential relations between the chironomid community and toxic contaminants in the Toce River (Northern Italy), focusing on a river stretch characterized by a gradient of dichloro-diphenyl-trichloroethane (DDT), mercury (Hg), and arsenic (As) industrial legacy contamination in conditions of relative uniformity of natural factors such as distance from the source, width, depth, and discharge. A DDT factory equipped with a mercury-cell chlor-alkali plant, located close to the riverbank, caused heavy DDT and Hg contamination in water and sediments during the last century (ca. 1915–1996). Additionally, documentation indicates arsenopyrite roasting to produce sulfuric acid in the industry, leading to As enrichment into the Toce [17]. Pollutant concentrations in different environmental compartments (sediments, biota) of this river are constantly monitored by the International Commission for the Protection of the Italian-Swiss Waters (CIPAIS; https://www.cipais.org/web/, accessed on 10 January 2024). Data indicate that contamination is slowly decreasing due to the natural recovery determined by the transport of uncontaminated sediments from upstream [18]. At the river mouth, total DDT concentrations decreased from 142 µg kg^−1^ normalized to 1% organic carbon (1% OC) in 2001 to 3.2 µg kg^−1^ 1% OC in 2018; Hg shifted from 949 µg kg^−1^ dry weight (d.w.) in 2008 to 68 µg kg^−1^ d.w. in 2018; As decreased from 48 mg kg^−1^ d.w. in 2008 to 17 mg kg^−1^ d.w. in 2018 [18], but concentrations in sediments and in the aquatic biota still remain above background levels [19,20].

The response of the entire benthic macroinvertebrate community in this river section was analyzed in 2014 [17]. Partial Redundancy Analysis (RDA) revealed that 5% of the total variance was associated with sediment contamination. Different biotic metrics were calculated, including non-stressor-specific metrics such as the Multimetric Intercalibration Index STAR_ICMi [21] and stressor-specific metrics such as Species at Risk for pesticides (SPEARpesticide [22]) and mean Sensitivity to Hg (SHg [23]), but significant differences between communities upstream and downstream of the factory were not emphasized.

In this study, we have furthered the taxonomic identification of chironomids to the most detailed level possible, aiming to understand if this species-rich group may exhibit relationships with residual contamination. According to published literature, these organisms may be sensitive to DDT, Hg, and As. DDT exposure is known to affect *Chironomus* emergence, mortality, reproduction, growth, and sex ratio [24,25]. Exposure to *Chironomus riparius* to Hg has been reported to reduce growth and emergence, delay development time, and decrease activity of the larvae [10], and increase morphological deformities [26]. The latter effect is commonly observed also with exposure to other trace elements, such as As [7,27]. However, community-level responses have rarely been reported. For example, Diggins and Steward [28] showed reduced richness and densities at increasing metal contamination levels, with *Procladius*, *Chironomus*, and *Cricotopus* sp. as the most tolerant taxa. Therefore, the ultimate objectives of this study are to assess the potential toxic effect of residual contamination on chironomid taxa assemblages and identify potential indicators of DDT, Hg, and As pollution.

## 2. Materials and Methods

### 2.1. Study Area

**Figure 1:**
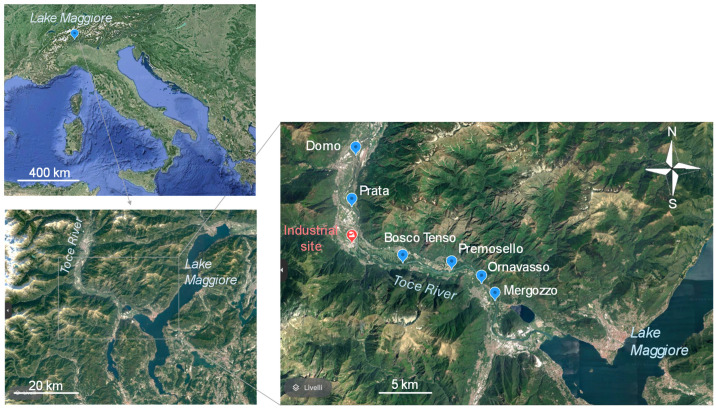
Map of the Toce River (Piedmont Region, Northern Italy) with the location of sampling stations (blue markers) and of the industrial site (red marker).

The Toce River is 84 km in length and is one of the primary tributaries of Lake Maggiore, situated along the boundary between Italy and Switzerland. Flowing through the Ossola Valley in the Central-Western Alps of the Piedmont Region, Northern Italy, the river exhibits an average annual flow of approximately 65 m^3^ s^−1^ (Figure 1). Within its catchment area of 1600 km^2^, natural land use predominates (92%), while urban and industrial areas only account for 2% and 0.4%, respectively [18].

The river section selected for this study is approximately 23 km long, with the most upstream station (Domo) located 8.6 km from the industrial site and the furthest downstream site (Mergozzo) situated 14.4 km away. This particular river stretch was selected due to its relatively uniform hydro-morphological characteristics, classified as WFD intercalibration type R-A2 and Italian river type 01SS4G, i.e., it belongs to the Western Alps hydroecoregion, situated at elevations ranging between 500 and 1000 m above sea level, characterized by runoff origins, a nival-glacial flow regime, siliceous substrate, and large size (distance from the source ranging between 75 and 150 km). Based on river discharge, this stretch comprises two distinct water bodies: type G1 upstream from Pieve Vergonte (average annual flow at Domo site: 32 m^3^ s^−1^) and type G2 downstream (average annual flow at Ornavasso site: 55 m^3^ s^−1^), influenced by the inflow of two large water channels (data provided by the Environmental Protection Agency of Piedmont Region, ARPA Piemonte, www.arpa.piemonte.it, accessed on 10 January 2024). The riverbed width ranges from 40 to 60 m, with a maximum depth exceeding 1.5 m.

Background values were estimated as 0.044 ± 0. 026 mg kg^−1^ d.w. for Hg and 34.3 ± 3.0 mg kg^−1^ d.w. for As in river sediments [29]. The relatively high geological background for As is due to the presence of arsenopyrite formations [30].

Six sampling sites were selected (Figure 1): two stations located upstream from the industrial site, namely Domo and Prata (8.6 km and 3.4 km from the industrial site, respectively); and four downstream stations, namely Bosco Tenso, Premosello, Ornavasso, and Mergozzo (3.7, 8.7, 13.1, and 14.4 km from the industrial site, respectively).

### 2.2. Insect Collection

Chironomids were collected in April, July, and October 2014 at all six stations and in February 2019 at all stations except Mergozzo. This latter sampling campaign was aimed at increasing the number of samples for statistical analyses and to account for potential interannual variations in the chironomid community and/or contaminant concentrations.

Sampling of chironomids followed the Italian standard protocol for benthic macroinvertebrate community [21], employing a multihabitat proportional method. Within each site (a 50 m long river stretch), a depositional area (pool mesohabitat) and an erosional area (riffle mesohabitat) were visually identified, where possible. Pools are characterized by finer substrates, greater depth, and lower flow velocity compared to riffles. Based on dominant substrates and flow types, different microhabitat types were identified within each of the two areas: silt (<63 µm grain size), sand (63 µm–2 mm grain size), microlithal (stones with 2–6 cm length), mesolithal (6–20 cm), macrolithal (20–40 cm), coarse particulate organic matter (CPOM), fine particulate organic matter (FPOM), algae, aquatic macrophytes, parts of terrestrial plants (Tp), and submerged dead wood (xylal) (Table S1). Ten replicate units were collected within both the pool and riffle, based on the relative occurrence of each microhabitat type [21]. Each replicate unit consisted of a 0.1 m^2^ area and was sampled using a Surber net (300 µm mesh size). In total, 114 quantitative samples were collected.

Additionally, drift nets were positioned at each site for at least one hour to collect pupal exuviae and floating adults. These qualitative samples were used solely to achieve a more precise taxonomic identification of taxa and were not included in statistical analyses.

Samples were preserved in 70% ethanol and sorted in the laboratory under a stereomicroscope. Chironomids were mounted onto slides and identified under microscope magnification to the most detailed taxonomic level possible, i.e., genus, species group, or species, following taxonomic keys [31,32,33]. Mature larvae of *Chironomus* were identified to species level by examining the karyotype of polytene chromosomes extracted from salivary glands, following the method described by Bettinetti et al. [34].

### 2.3. Water Quality and Sediment Chemical Analyses

Sediment sampling was carried out at the same sites and dates as the chironomid samplings. Different sub-samples were collected at each site using a metal spoon and mixed to obtain a representative sample. The sediments were preserved in acid-washed dark glass bottles at 4 °C until freeze-drying (72 h at 0.2 mbar and −45 °C; Telstar LyoQuest system, Telstar, Élancour, France). Sediments were sieved to separate the finest fraction (<63 µm grain size) for chemical analyses.

Concentrations of DDT (sum) and trace elements (Hg, As, Cd, Cu, Ni, and Pb) in sediments collected in 2014 were analyzed and published in Marziali et al. [17], and concentrations of DDT and Hg in 2019 samples were reported in CIPAIS [35], thus these data were used for statistical analyses. Concentrations of the other trace elements (As, Cd, Cu, Ni, and Pb) in 2019 samples are new original data presented in this work. For this analysis, the following procedure was followed: aliquots of 150–200 mg of freeze-dried sediments (<63 µm grain size fraction) were homogenized with a ball mill (Retsch MM2000, Retsch Technology GmbH, Haan, Germany) and mineralized in closed pressurized Teflon vessels with 6 mL of concentrated HNO_3_ and 2 mL of ultrapure water using a microwave system (Preekem Excel, Fulltech Instruments, Arenato, Rome, Italy). Solutions were diluted to 50 mL with ultrapure water. After centrifugation, quantification was carried out by Inductively Coupled Plasma-Optical Emission Spectrometry (ICP-OES) (iCap7200 Duo, Thermo Fisher Scientific, Rodano, Milan, Italy). For quality assurance, multielement standard solutions at 20 µg L^−1^ and 100 µg L^−1^ were analyzed every 10 samples, with recoveries ranging between 93 and 110% for all elements. Blank values were always below the Limit of Detection (LOD). The certified reference material GBW07305 sediment powder from the National Standard Centre of China was analyzed, with recoveries ranging between 85% and 99% for all elements. Analyses were run in triplicate, obtaining relative standard deviations ≤ 10%.

Total organic carbon (OC) in sediments was determined in 0.5 g d.w. sample aliquots by back-titration after oxidation with potassium dichromate in the presence of sulfuric acid, following the Walkley-Black procedure [36].

During each sampling, water depth was measured with a graduated rod, water velocity was estimated with a flow probe (PCE, PCE Italia s.r.l., Lucca, Italy), and water parameters were measured using field multiprobes (Radiometer Analytical, Hach, Milan, Italy): pH, water temperature, water conductivity, and percent oxygen saturation (Table 1). The dominant flow type was visually estimated as smooth, rippled, unbroken standing waves, broken standing waves, or chute.

Regarding water quality analysis, data on total alkalinity, nitrates (N-NO_3_), ammonia (N-NH_4_), total phosphorous and dissolved DDT (sum of DDT, DDE, and DDE), and trace elements were provided by ARPA Piemonte [14], with site and sampling season used as criteria to match chemical data with chironomid samplings (Table 1). Those data were included to test the potential influence of water parameters on chironomids. Dissolved DDT, Hg, and As were mostly below the LOD (Table 1), therefore they were not considered in the data analysis.

### 2.4. Data Analysis

A multivariate analysis was conducted to examine the relationship between chironomid community composition and environmental factors. The environmental variables considered are outlined in Table 1 and include altitude, distance from the source, mean water depth, current velocity, water temperature, alkalinity, water conductivity, pH, oxygen saturation, ammonia, nitrates, total phosphorous, dominant flow type (coded as follows: 1 = smooth, 2 = rippled, 3 = unbroken standing waves, 4 = broken standing waves, 5 = chute), percent fine fraction (<63 μm grain size) in whole sediments, organic carbon in sediments, concentration of micropollutants and trace metals in sediments, including DDT, As, Hg, Cu, Cd, Ni, and Pb. Additionally, two mesohabitats (pool and riffle), each with different microhabitat types (silt, sand, microlithal, mesolithal, macrolithal, CPOM, FPOM, algae, aquatic macrophytes, Tp, and xylal), were included, totaling 114 samples. In terms of species, 32 taxa identified to genus or species level were included.

The gradient length calculated using Detrended Correspondence Analysis (DECORANA) suggested a bimodal response as more appropriate than a linear one. Therefore, Canonical Correspondence Analysis (CCA) was preferred over Redundancy Analysis (RDA) [37]. The “formula interface” method was chosen for analysis [38,39]. A nXp matrix, comprising n (=114) rows (i.e., microhabitat samplings) and p (=32) columns (chironomid taxa), and a nZs matrix, including the same rows and s (=19) columns (environmental variables), were analyzed. The nXp values were log(x + 1) transformed before calculation.

A forward selection of environmental variables was run to test reduced models containing only the variables contributing most to total variance. With this method, collinear variables were removed. Subsequently, partial Canonical Correspondence Analysis (pCCA) [38,39] was carried out, running a CCA analysis with a species data matrix nXp explained by an environmental data matrix nZs, which constitutes the constrained variables in the presence of conditioning variables nWt (covariates). This pCCA aimed to assess the influence of sediment contamination (DDT, Hg, and As concentrations) on overall community variability. Subsequently, a variation partitioning test was carried out. With this method, the variation bound to sediment contamination was calculated by removing the influence of other variables.

The significance of CCA, pCCA, and variation partitioning was tested by running a Monte Carlo simulation with 999 permutations.

A Self-Organizing Map (SOM) was trained based on the nXp matrix [40]. SOM approaches k-mean clustering in aggregating sites in units and has the advantage that it is not affected by outliers [41,42]. SOM creates a map in which sites are plotted according to similarities in species composition: sites with similar species assemblages are aggregated in clusters of cells. SOM allows a simple representation of sites and species in two dimensions and can be used to easily represent the response of single taxa to target parameters. In fact, SOM analysis was trained as a Supervised Self-Organizing Map (SSOM) [43]. In SSOM, an external variable is included to guide the clustering. Different SSOMs were trained, each including a different column of the nZs matrix, representing an environmental variable or factor. In the present analysis, the site maps were created by dividing the values of each environmental variable into classes to create a factor with different levels, represented by different colors. Cells with the same environmental factor level and similar species composition were clustered together. The empty cells emphasize the distance between clusters (filled cells). Species maps were produced based on codebook values. The relative abundance of each species was figured out on the map. SOM map size was selected considering quantization (QE) and topographic error (TE). QE is a measure of the average distance between the data points and the map nodes to which they are mapped, with smaller values indicating a better fit. TE is a measure of how well the structure of the input space is modeled by the map [44].

All data analyses were carried out in the R environment (R version 4.3.2, 2023-10-31 ucrt) [38,45] supplemented by the following additional packages: vegan [46], adespatial, gclus, cluster, Hmisc, labdsv, pvclust, kohonen [47], awesome [48], plotrix, and RColorBrewer.

## 3. Results

The ranges of the environmental parameters measured at the sampling sites are presented in Table 1. The target parameters included DDT, showing values in sediments comprised between 0.4 and 15.7 µg kg^−1^ 1% OC; Hg, comprised between 20 and 197 µg kg^−1^ d.w.; and As, ranging between 3.6 and 55.7 mg kg^−1^ d.w. (Table 1). Concentrations of contaminants in sediments collected at the downstream sites were significantly higher than those at the upstream sites (*t*-test, *p* < 0.05; Table 2).

**Table 1:** Environmental parameters used in analyses with range and mean values. Abbreviations used in Figures and Tables are also reported.

Compartment	Parameter	Unit	Abbreviation	Min	Max	Mean
	Altitude	m a.s.l.	alt	196	236	217
	Distance from the source	km	dist	49	72	59
Sediments	Fine sediments	%	sed	0.2	27	7
	Organic carbon	% d.w.	sostorg	0.47	3.7	1.4
	DDT	µg kg^−1^ 1% OC	DDT	0.4	15.7	6.2
	Hg	mg kg^−1^ d.w.	Hg	0.02	0.197	0.081
	As	mg kg^−1^ d.w.	As	3.6	55.7	16.6
	Cd	mg kg^−1^ d.w.	Cd	0.1	0.3	0.2
	Cu	mg kg^−1^ d.w.	Cu	13.7	55.9	28.7
	Ni	mg kg^−1^ d.w.	Ni	12.3	47.2	31.5
	Pb	mg kg^−1^ d.w.	Pb	8.7	17.7	13.4
Water	Sampling depth	cm	depth	5	50	24
	Current velocity	cm s^−1^	velcorr	1	100	20
	Water temperature	°C	temp	7	15.7	10.9
	Conductivity	µS cm^−1^	cond	122	269	181
	Alkalinity	mg L^−1^ CaCO_3_	alcal	36	102	68
	pH	pH unit	pH	7.1	8	7.5
	N-NO_3_	mg L^−1^	NO_3_	<0.01	0.5	0.4
	N-NH_4_	mg L^−1^	NH_4_	<0.01	0.03	0.015
	dissolved DDT	ng L^−1^	-	<2	<2	<2
	dissolved Hg	ng L^−1^	-	<20	<20	<20
	dissolved As	µg L^−1^	-	<3	4	<3
	Total Phosphorus	mg L^−1^	TP	<0.005	<0.005	<0.005
	Oxygen saturation	%	O_2_	96	124	102

A total of 32 taxa were collected using the multihabitat proportional method and were included in the multivariate analyses (Table 3). The most frequent species were *Micropsectra atrofasciata*, *Microtendipes pedellus*, *Polypedilum* spp., *Tvetenia* spp., *Odontomesa fulva*, and *Macropelopia* spp.

A comprehensive list of species collected in the Toce River, considering also samples obtained using drift nets, is presented in Table S2.

Correlations between environmental parameters and species are presented in Table S3.

**Figure 2:**
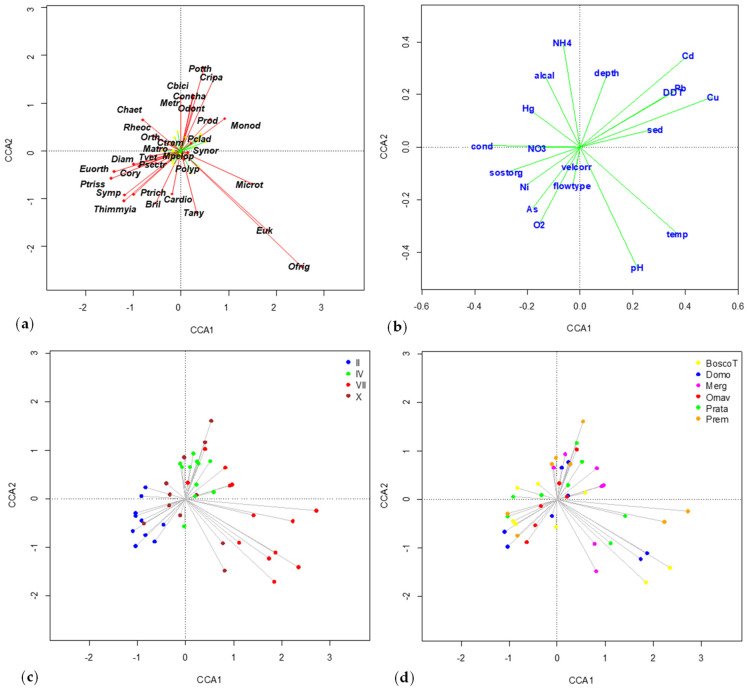
Results of CCA, including all variables. Plot of the first two axes with scores of (**a**) species and (**b**) environmental variables. Cases are colored according to: (**c**) sampling month; (**d**) station. Abbreviations of variable and species names are reported in Table 1 and Table 3.

A preliminary DECORANA analysis yielded axis lengths of 5.060, 4.762, 3.826, and 4.010 in the first four axes, respectively, suggesting a unimodal species-environment relationship. According to the CCA analysis, the chironomid community exhibited a strong correlation with the 19 environmental factors included in the analysis, explaining 32% of the total inertia (Table S4a). The scores of species and environmental variables for the first two canonical axes are depicted in Figure 2a,b (Tables S5 and S6a).

The CCA highlighted a spatio-temporal gradient (Figure 2c,d). The temporal gradient, evident along the first axis, reflected seasonality, with February samples distinct from July samples, consistent with variations in water temperature values (Figure 2c, Table S5). The spatial gradient manifested as an upstream-downstream ordination along the second axis, separating sites based on increasing depth (Figure 2d, Table S5). However, this trend was confounded by the predominant seasonal gradient. The species driving ordination were *Orthocladius frigidus* and *Eukiefferiella* spp., which clearly prevailed in the upstream stations, except in February, when *Diamesa* spp. and *Orthocladius* (*Euorthocladius*) spp. dominated (Table S6a). *C. riparius* predominated in October. *Potthastia longimanus*, *Conchapelopia pallidula*, *Cricotopus bicinctus*, and *C. riparius* showed high scores on the second axis with no clear spatial distribution (Table S6a).

The contaminant DDT, along with the trace elements Cu, Cd, and Pb, were associated with percent fine sediments, while As and Ni correlated with organic carbon (Figure 2b) (Tables S3 and S5). Conversely, Hg followed a different pattern, likely influenced by the seasonal gradient.

The stepwise forward analysis conducted to reduce the number of environmental variables identified water temperature, Pb, and flow type as the most significant variables (i.e., with the highest Variance Inflation Factor) to be included in the model, with an adjusted R squared of 0.066, 0.061, and 0.025, respectively. These variables confirmed the spatio-temporal gradient, with a seasonal gradient (water temperature) prevailing on the spatial one (Pb). Temperature was associated with temporal factors (sampling month), Pb was correlated with spatial factors (altitude and distance from the source), and flow type was associated with current velocity (Figure 2, Table S3). This trace element, as well as Cd, Cu, and Ni, were proved to be mostly bound to geogenic origin [18], with slight differences between concentrations at upstream and downstream sites (Table 2). For this reason, they were considered natural variables.

**Figure 3:**
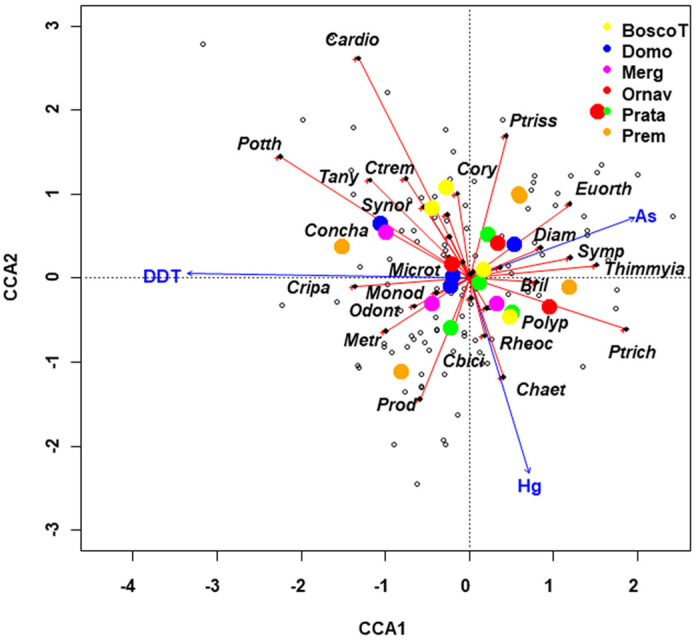
Partial CCA, including DDT, Hg, and As as constrained variables, with water temperature (temp), Pb concentration in sediments, and flow type as conditioning variables. Sampling stations (centroids) are plotted with different colors. Abbreviations of species and variable names are reported in Table 1 and Table 3.

Partial CCA was subsequently performed (Table S4b). Variables measuring sediment contamination (DDT, Hg, and As concentrations) were included as constrained variables in the nZs matrix, while temperature, Pb, and flow type were included as conditioning variables in the nWt matrix (Figure 3). The analysis aimed to determine the extent to which the constrained variables explained the chironomid species pattern when the effect of the natural variables was removed. The results indicated that constrained variables explained 5.2% of total inertia, while conditioning ones accounted for 3.7% (Table S4b).

Partial CCA allowed to separate the upstream sites Domo and Prata, which clustered near the center of the plot, from the contaminated stations, namely Premosello, Bosco Tenso, Ornavasso, and Mergozzo, which were positioned at some distance from the center. DDT and As exerted opposite influences on the first axis, while Hg was correlated with the second axis (Figure 3, Table S7).

Some species were positioned far from the center of the graph and exhibited an inverse correlation with DDT, Hg, and As, respectively, indicating sensitivity to the presence of these toxicants (Figure 3, Tables S6b and S7). *Cricotopus (Paratrichocladius) rufiventris*, *Thienemannimyia* sp., *Sympotthastia spinifera,* and *Diamesa* spp. showed an inverse correlation with DDT; *Cardiocladius* sp. and *P. longimanus* with Hg; *C. riparius*, *Prodiamesa olivacea*, *Chaetocladius* spp., *Orthocladius (Euorthocladius)* spp., and *Paratrissocladius excerptus* with As. Conversely, factor score values indicated tolerance of *C. riparius* to DDT and of *P. olivacea* to Hg (Figure 3, Tables S6b and S7).

**Figure 4:**
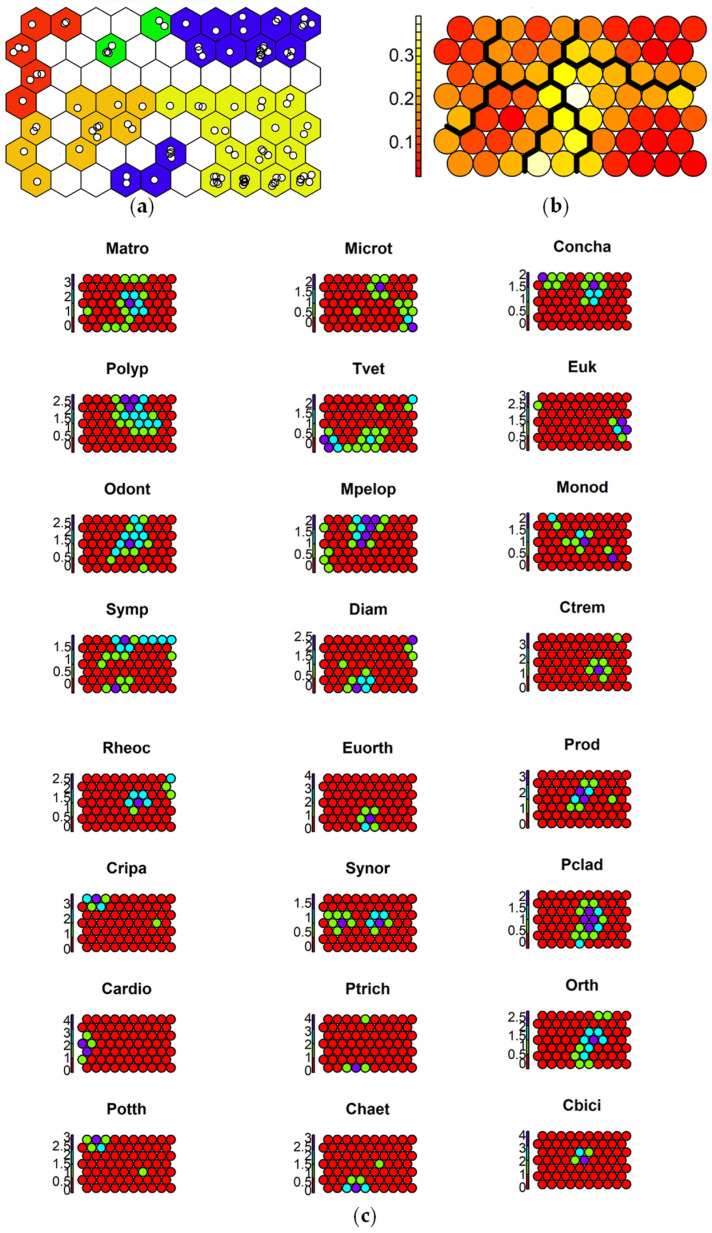
Results of SSOM analysis using DDTsum as an ordering external factor: (**a**) sites mapped in 5 clusters, with different colors: <3 µg kg^−1^ 1%OC (blue cells), 3–4.5 µg kg^−1^ 1%OC (green cells), 4.5–7.5 µg kg^−1^ 1%OC (yellow cells), 7.5–12 µg kg^−1^ 1%OC (orange cells), >12 µg kg^−1^ 1%OC (red cells); (**b**) distances between clusters: high distances in yellow, low distances in red; (**c**) species graphs representing the different abundances of a species in the cells (values on y axis represent log_10_-tranformed abundance): very abundant (purple cells), abundant (cyan cells), present (green cells), absent (red cells). Abbreviations of species names are reported in Table 3.

**Figure 5:**
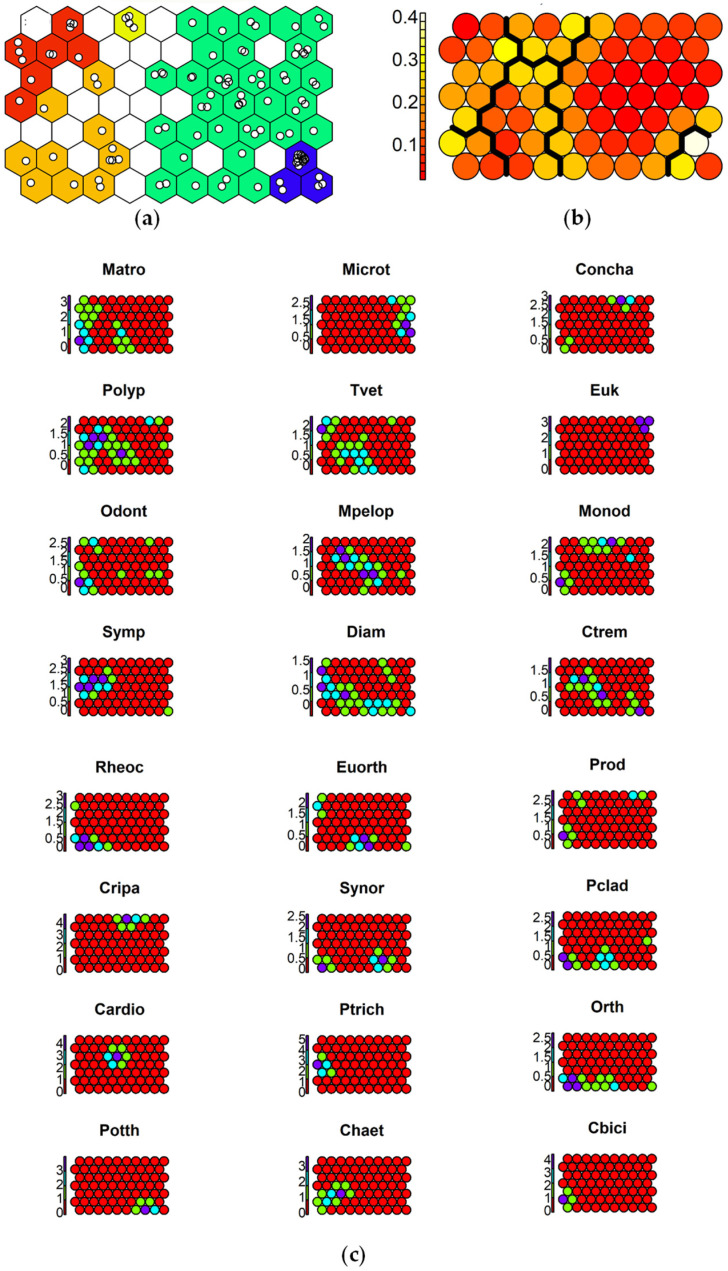
Results of SSOM analysis using Hg as an ordering external factor: (**a**) sites mapped in 5 clusters, with different colors: <40 µg kg^−1^ d.w. (blue cells), 40–60 µg kg^−1^ d.w. (green cells), 60–100 µg kg^−1^ d.w. (yellow cells), 100–160 µg kg^−1^ d.w. (orange cells), >160 µg kg^−1^ d.w. (red cells); (**b**) distances between clusters: high distances in yellow, low distances in red; (**c**) species graphs representing the different abundances of a species in the cells (values on y axis represent log_10_-tranformed abundance): very abundant (purple cells), abundant (cyan cells), present (green cells), absent (red cells). Abbreviations of species names are reported in Table 3.

**Figure 6:**
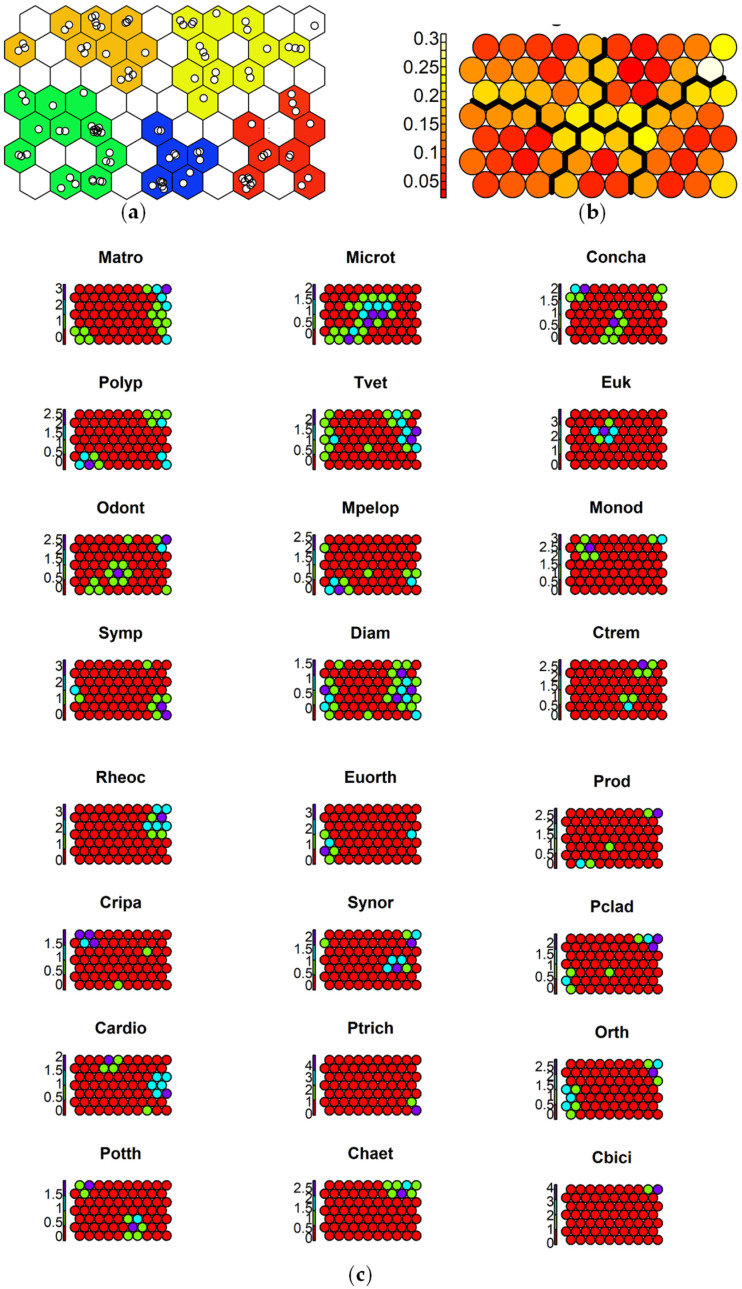
Results of SSOM analysis using As as an ordering external factor: (**a**) sites mapped in 5 clusters, with different colors: <8 mg kg^−1^ d.w. (blue cells), 8–10 mg kg^−1^ d.w. (green cells), 10–16 mg kg^−1^ d.w. (yellow cells), 16–25 mg kg^−1^ d.w. (orange cells), >25 mg kg^−1^ d.w. (red cells); (**b**) distances between clusters: high distances in yellow, low distances in red; (**c**) species graphs representing the different abundances of a species in the cells (values on y axis represent log_10_-tranformed abundance): very abundant (purple cells), abundant (cyan cells), present (green cells), absent (red cells). Abbreviations of species names are reported in Table 3.

To aid in the interpretation of the response of each species to the toxicants, SSOM maps were used. The selected map size was 10 × 7 cells, which optimized quantization (QE) and topographic (TE) errors for the 70-cell map size carried out with 1000 iterations (Table S8). The species responses to contamination are reported in Figure 4, Figure 5 and Figure 6.

Species maps showed that almost all taxa seem to avoid the sites with the highest DDT concentrations, while they could tolerate moderate DDT contamination (Figure 4). Only *C. riparius* and *P. longimanus* showed the highest abundance at sites with the highest DDT levels. Some taxa, such as *Diamesa* spp., *S. spinifera*, and *P. olivacea*, were mostly abundant at sites with the lowest DDT levels, suggesting sensitivity to this toxicant.

Species tolerant to high Hg concentrations included *M. atrofasciata*, *Polypedilum* spp., *O. fulva*, *Rheocricotopus* spp., *C. bicinctus*, and *P. olivacea*, while only *P. longimanus* avoided sites with the highest Hg concentrations (Figure 5).

Arsenic had a limited influence on species distribution. Only *M. pedellus* and *Orthocladius (Euorthocladius)* spp. avoided sites with the highest As concentrations. Conversely, *S. spinifera* demonstrated tolerance to the toxicant, being most abundant at sites with the highest As concentrations (Figure 6).

Species responses to other variables are reported in Supplementary material: responses to the longitudinal gradient of the river (distance from the source) are reported in Figure S1; to seasonality (sampling month and water temperature) in Figures S2 and S3; to habitat characteristics (mesohabitat and microhabitat type) in Figures S4 and S5; to hydrological factors (flow type, current velocity, and depth) in Figures S10–S12; to physical-chemical parameters of water (conductivity, oxygen saturation) in Figures S13 and S14; to organic carbon in sediments in Figure S15. These responses may be used to derive the ecological traits of species.

## 4. Discussion

The values of contaminants (DDT, Hg, and As) in sediments downstream of the industrial plant are typical of legacy contamination [19,20,49,50]. Despite covering a relatively narrow range, concentrations exceeded the consensus-based Threshold Effect Concentrations (cb-TECs), indicating thresholds below which toxic effects for benthic invertebrates are not expected (5.28 µg kg^−1^ 1%OC for total DDT, 180 µg kg^−1^ d.w. for Hg, 9.79 mg kg^−1^ d.w. for As) [51], showing maxima up to 15.7 µg kg^−1^ 1%OC for DDT, 197 µg kg^−1^ d.w. for Hg, and 55.7 mg kg^−1^ d.w. for As. Similar values of DDT and Hg are often observed in freshwater ecosystems, even in the absence of local anthropogenic sources, due to the atmospheric long-distance transport of these contaminants [52,53]. For As, the main source in freshwater is generally geogenic [30,54]. However, in the case of the Toce River, contributions from past anthropogenic activities are reported [17,55] and downstream values exceed the background value of 34.3 ± 3.0 mg kg^−1^ d.w. [29], reaching concentrations above the consensus-based Probable Effect Concentration (cb-PEC, 33 mg kg^−1^ d.w.), which is the threshold above which effects on benthic organisms are expected [51].

The Toce River hosts species-rich benthic invertebrate communities [17], as confirmed by chironomid taxa assemblages, which comprised a total of 56 taxa resulting from the analysis of both Surber net and drift net samples (Table S2). This number aligns with taxa richness values obtained in other Italian lowland rivers, such as 56 chironomid taxa in the Adda River (Northern Italy, [56,57]), 68 taxa in the Adige River (Northern Italy [58]), and 79 taxa in Southern rivers (Cilento National Park [59]). The presence of many species, including some sensitive to anthropogenic alterations such as *O. fulva* and *S. spinifera*, offers the potential to identify indicators of residual toxic contamination.

The CCA results emphasized that chironomid taxa assemblages were primarily structured by temporal (seasonal) factors rather than the spatial (upstream-downstream) gradient. In fact, species separation n was more evident when grouping sites by sampling month rather than by distance from the source.

Temporal gradients are critical in structuring chironomid communities due to their association with the adult emergence seasonality [60,61]. Most species show their optimum for emerge in winter, spring, summer, or, more rarely, autumn. In this study, we considered different sampling seasons with the aim of collecting a representative set of species. Additionally, samples were collected in different years (2014 and 2019) to account for potential interannual variability related to climatic and hydrological factors [62]. This may explain the strong temporal-seasonal gradient observed.

The spatial gradient within a watershed is generally related to altitude, distance from the source, and it may also be influenced by water depth, water temperature, dissolved oxygen, current velocity, substrate grain size, substrate type, etc. These variables significantly influence chironomid species composition [1,2,15]. In our study, the river stretch considered was selected to minimize the influence of these parameters, with any spatial gradient expected to be primarily bound to the contamination gradient.

To analyze the response of chironomids to contamination, it was necessary to remove the effects of natural spatio-temporal factors. Partial CCA (pCCA) showed that only a small proportion of the total variance could be attributed to pollutants (5.2%). Chironomids were generally tolerant to a wide range of conditions, and only in some cases was it possible to detect a differential response among species.

With the aim of SSOM maps, the distribution of species according to toxicant concentrations was analyzed. The prevalence of *C. riparius* at sites with higher DDT concentrations agrees with the identification of this species as one of the most tolerant [63]. Similarly, the prevalence of *M. atrofasciata* at the sites with the highest Hg values confirms the tolerance of this species [3]. *C. pallidula* and *Polypedilum* spp. (including *Polypedilum nubeculosum* and *Polypedilum laetum*) were confirmed as tolerant species [3,5]. Conversely, *O. fulva, S. spinifera*, and *P. longimanus* appeared less tolerant than previously thought [4]. In fact, *O. fulva*, as well as *M. pedellus*, showed maxima at sites with the lowest As concentrations. *P. longimanus* was sensitive to Hg and to DDT. *S. spinifera* avoided DDT-contaminated sites, as did *Diamesa* spp. (including *Diamesa zernyi* and *Diamesa tonsa*), confirming them to be sensitive taxa [4].

The results also allowed for a preliminary outline of some ecological traits of the most frequent and/or peculiar species, as reported in Table 4. The statistical significance of these traits cannot always be tested due to the need for a larger dataset. However, these descriptions may serve as a basis for future studies aimed at using chironomids as indicators of specific habitat alterations [64].

## 5. Conclusions

Chironomids are primarily responsive to various natural factors, including season, water temperature, flow type, and substrate type. However, certain taxa may exhibit sensitivity to contaminants that accumulate in river sediments, although this finding requires further investigation. This study represents a preliminary effort to analyze the response of chironomid communities to contamination levels commonly observed in freshwater ecosystems.

## Figures and Tables

**Table 2:** Comparison between concentrations of DDT and trace elements (mean ± standard deviation) in sediments collected at sites upstream and downstream of the industrial site (*t*-test, *p*-value).

Contaminant	Unit	Upstream Sites	Downstream Sites	*p*-Value
DDT	µg kg^−1^ 1% OC	4.0 ± 3.1	7.5 ± 3.9	<0.0001
Hg	mg kg^−1^ d.w.	0.05 ± 0.02	0.10 ± 0.05	<0.0001
As	mg kg^−1^ d.w.	7.2 ± 3.0	22.1 ± 9.8	<0.0001
Cd	mg kg^−1^ d.w.	0.15 ± 0.04	0.17 ± 0.04	0.111
Cu	mg kg^−1^ d.w.	31.2 ± 12.9	27.2 ± 8.1	0.044
Ni	mg kg^−1^ d.w.	37.4 ± 5.9	28.0 ± 6.2	<0.0001
Pb	mg kg^−1^ d.w.	12.6 ± 2.8	13.8 ± 2.2	0.015

**Table 3:** List of chironomid taxa collected in the Toce River using the multihabitat proportional method, with abbreviations used in Figures, density, and frequency (i.e., percent of samples where the species was observed on a total of 114 samples).

Taxa	Authors	Abbreviation	Density (ind m^−2^)	Frequency (%)
*Micropsectra atrofasciata*	(Kieffer, 1911)	Matro	177	35
*Microtendipes pedellus*	(De Geer, 1776)	Microt	169	31
*Polypedilum* spp.	Kieffer, 1912	Polyp	321	31
*Tvetenia* spp.	Kieffer, 1922	Tvet	222	31
*Odontomesa fulva*	(Kieffer, 1919)	Odont	2026	29
*Macropelopia* spp.	Thienemann, 1916	Mpelop	136	22
*Sympotthastia spinifera*	Serra-Tosio, 1968	Symp	364	22
*Diamesa* spp.	Meigen, 1835	Diam	160	24
*Conchapelopia pallidula*	(Meigen, 1818)	Concha	81	16
*Prodiamesa olivacea*	(Meigen, 1818)	Prod	102	16
*Eukiefferiella* spp.	Thienemann, 1926	Euk	229	23
*Paracladopelma* spp.	Harnish, 1923	Pclad	38	13
*Monodiamesa batyphila*	(Kieffer, 1918)	Monod	91	12
*Orthocladius (Orthocladius)* spp.	Van der Wulp, 1874	Orth	31	12
*Cricotopus (Cricotopus) tremulus*	(Linnaeus, 1756)	Ctrem	42	11
*Cricotopus (Cricotopus) bicinctus*	(Meigen, 1818)	Cbici	168	10
*Rheocricotopus* spp.	Brundin, 1956	Rheoc	13	10
*Orthocladius (Euorthocladius)* spp.	Thienemann, 1935	Euorth	49	10
*Chironomus riparius*	Meigen, 1804	Cripa	59	7
*Synorthocladius semivirens*	(Kieffer, 1909)	Synor	29	6
*Cardiocladius* sp.	Kieffer, 1912	Cardio	12	5
*Cricotopus (Paratrichocladius) rufiventris*	(Meigen, 1830)	Ptrich	35	5
*Potthastia longimanus*	(Kieffer, 1922)	Potth	27	5
*Chaetocladius* spp.	Kieffer, 1911	Chaet	23	4
*Thienemannimyia* sp.	Fittkau, 1957	Thimmyia	45	4
*Corynoneura* spp.	Winnertz, 1846	Cory	6	4
*Metriocnemus* spp.	van der Wulp, 1874	Metr	3	3
*Brillia* spp.	Kieffer, 1913	Bril	9	3
*Orthocladius (Mesorthocladius) frigidus*	(Zetterstedt, 1838)	Ofrig	11	2
*Psectrocladius* sp.	Kieffer, 1906	Psectr	5	2
Tanytarsini spp.	-	Tany	2	2
*Paratrissocladius excerptus*	(Walker, 1856)	Ptriss	2	1

**Table 4:** Ecological traits of the most typical species collected in the Toce River.

Taxa	Ecological Traits
*Macropelopia* spp.	The genus prefers waters with higher oxygen saturation and moderate water flow. No particular sensitivity to toxicants was observed. It is a predator of other chironomid larvae, or Oligochaetes [5].
*Conchapelopia pallidula*	The species prevails at the downstream stations in the summer. It prefers the pool habitat. It shows no flow type preference and no sensitivity to toxicants in the Toce River. It is a common predator of other chironomid larvae and Oligochaetes [5].
*Diamesa* spp.	The taxon includes *Diamesa zernyi* and *Diamesa tonsa*. It prefers running waters with high flow velocity and turbulence, such as upstream riffle habitats with lower water temperatures. It tolerates a moderate organic carbon concentration in sediments and high Hg levels, while it is mostly absent at sites with higher DDT concentrations.
*Potthastia longimanus*	This species prefers pool microhabitat, silty sediments, broken standing wave flow type, moderate organic matter content in sediments, and high DDT concentrations, while it is moslty absent at sites with higher Hg concentrations.
*Sympotthastia spinifera*	Abundant in February, this species is rare in October and April. It prefers pools rich in submerged macrophytes, but it is present at all microhabitat types and stations. It tolerates high organic carbon content (>3.2%) and is present at sites most contaminated by As and Hg, while it is mostly absent at DDT-contaminated sites.
*Odontomesa fulva*	Abundant in April, it is rare in October and February. It prefers pool areas with rippled flow type and sand and mesolithal microhabitats, tolerating organic carbon in sediments. It is present at all stations, including sites with higher Hg and DDT concentrations but lower As levels.

## Data Availability

Data regarding sediment contamination analysis and benthic invertebrates of the Toce River are published in the project reports regarding the analysis of contamination in Lake Maggiore, available at the website www.cipais.org/web (accessed on 10 January 2024).

## Supplementary Materials

The following supporting information can be downloaded at: https://www.mdpi.com/article/10.3390/insects15030148/s1, Table S1: List of microhabitats; Table S2: List of chironomid taxa collected in the Toce River; Table S3: Correlations between taxa and environmental parameters; Table S4: Inertia and eigenvalues of CCA and partial CCA axes; Table S5: Results of CCA: scores of environmental factors; Table S6: Scores of species according to CCA and partial CCA; Table S7: Results of pCCA: constraining variables; Table S8: Results of SOM: Quantization and Topographic errors; Figure S1: SSOM map according to distance from the source; Figure S2: SSOM map according to sampling month; Figure S3: SSOM map according to water temperature; Figure S4: SSOM map according to mesohabitat; Figure S5: SSOM map according to microhabitats; Figure S6: SSOM map according to Pb; Figure S7: SSOM map according to Ni; Figure S8: SSOM map according to Cd; Figure S9: SSOM map according to Cu; Figure S10: SSOM map according to flow type; Figure S11: SSOM map according to current velocity; Figure S12: SSOM map according to water depth; Figure S13: SSOM map according to water conductivity; Figure S14: SSOM map according to oxygen saturation; Figure S15: SSOM map according to organic carbon.
