# Supplementary material for: Response of Chironomidae (Diptera) to DDT, Mercury, and Arsenic Legacy Pollution in Sediments of the Toce River (Northern Italy)
Source: Insects. 2024 Feb 22;15(3):148. doi: 10.3390/insects15030148 (PMC10971021; doi:10.3390/insects15030148)
Supplement: Supplementary file 1 [file insects-15-00148-s001.zip › insects-2849782-supplementary.pdf]

Response of Chironomidae (Diptera) to DDT, Mercury, and Arsenic Legacy Pollution in Sediments of the Toce River (Northern Italy)

Laura Marziali [1,*], Niccolò Pirola [1], Alfredo Schiavon [1,2,†] and Bruno Rossaro [3]

[1] National Research Council-Water Research Institute (CNR-IRSA), Via del Mulino 19, 20861 Brugherio (MB) Italy; niccolo.pirola94@gmail.com (N.P.); alfredo.schiavon@igb-berlin.de(A.S.)
[2] Department of Biology, Chemistry, and Pharmacy, Freie Universität Berlin, Arnimallee 22, 14195 Berlin, Germany
[3] Department of Agricultural and Environmental Sciences (DISAA), University of Milan, Via Celoria 2, 20133 Milan (MI), Italy; bruno.rossaro@unimi.it
* Correspondence: laura.marziali@irsa.cnr.it; Tel. +39-03921694207
† Current address: Department of Ecohydrology, IGB Leibniz-Institute of Freshwater Ecology and Inland Fisheries, Müggelseedamm 310, 12587 Berlin, Germany.

Supplementary material

Table S1. List of microhabitat types, description, and relative occurrence in the Toce River.

**Microhabitat type**	**Description**	**Occurrence in the Toce River** (%)
mesolithal	6-20 cm cobbles	36
sand	sand fraction (63 µm -2 mm)	20
microlithal	2-6 cm stones	16
algae	algae	7
silt	fine fraction (< 63 µm)	6
FPOM	fine organic matter	6
aquatic macrophytes	including aquatic moss	4
macrolithal	20-40 cm cobbles	2
CPOM	dead leaves and plant twigs	2
Tp	part of living terrestrial plants	1
xylal	dead wood	0.2

Table S2. Taxonomic list of taxa collected in the Toce River (* taxa included in data analysis).

**Taxon**	**Author**	**Data analysis**
Procladius (Holotanypus) choreus	(Meigen,1804)	
Macropelopia spp.	Thienemann,1916	*
Thienemannimyia carnea	(Fabricius,1805)	*
Conchapelopia pallidula	(Meigen,1818)	*
Diamesa tonsa	(Walker,1856)	*
Diamesa zernyi	Edwards,1933	
Sympotthastia spinifera	Serra-Tosio,1968	*
Potthastia gaedii	(Meigen,1838)	*
Potthastia longimanus	(Kieffer,1922)	
Monodiamesa bathyphila	(Kieffer,1918)	*
Odontomesa fulva	(Kieffer,1919)	*
Prodiamesa olivacea	(Meigen,1818)	*
Prodiamesa rufovittata	Goetghebuer,1932	
Brillia bifida	(Kieffer,1909)	*
Brillia longifurca	Kieffer,1921	

Cardiocladius sp.	Kieffer,1912	*
Tvetenia bavarica	(Goetghebuer,1934)	
Tvetenia calvescens	(Edwards,1929)	*
Eukiefferiella dittmari	Lehmann,1972	
Eukiefferiella gracei	(Edwards,1929)	
Eukiefferiella ilkleyensis	(Edwards,1929)	
Eukiefferiella devonica	(Edwards,1929)	
Eukiefferiella minor	(Edwards,1929)	
Eukiefferiella claripennis	(Lundbeck,1898)	*
Eukiefferiella fuldensis	Lehmann,1972	
Psectrocladius (Psectrocladius) sordidellus	(Zetterstedt,1838)	*
Rheocricotopus (Psilocricotopus) glabricollis	(Meigen,1830)	
Rheocricotopus (Rheocricotopus) effusus	(Walker,1856)	
Rheocricotopus (Rheocricotopus) fuscipes	Kieffer,1909	*
Synorthocladius semivirens	(Kieffer,1909)	*
Orthocladius (Euorthocladius) rivicola	Kieffer,1921	*
Orthocladius (Euorthocladius) rivulorum	Kieffer,1909	
Orthocladius (Mesorthocladius) frigidus	(Zetterstedt,1838)	*
Orthocladius (Orthocladius) excavatus	Brundin, L., 1947	
Orthocladius (Orthocladius) glabripennis	(Goetghebuer,1921)	*
Orthocladius (Orthocladius) rhyacobius	Kieffer,1911	
Cricotopus (Paratrichocladius) rufiventris	(Meigen,1830)	*
Cricotopus (Cricotopus) tremulus	(Linnaeus,1756)	*
Cricotopus (Cricotopus) annulator	Goetghebuer,1927	
Cricotopus (Cricotopus) bicinctus	(Meigen,1818)	*
Cricotopus (Isocladius)	Kieffer,1909	
Genus sp. *(Oliveridia ?)*	Sæther,1980	
Metriocnemus spp.	van der Wulp,1874	*
Chaetocladius spp.	Kieffer,1911	*
Paratrissocladius excerptus	(Walker, 1856)	*
Thienemanniella sp.	Kieffer,1911	
Corynoneura scutellata	Winnertz,1846	*
Virgatanytarsus albisutus	(Santos-Abreu,1918)	
Paratanytarsus sp.	Thienemann & Bause,1913	*
Micropsectra atrofasciata	(Kieffer,1911)	*
Microtendipes pedellus	(De Geer,1776)	*
Polypedilum (Polypedilum) laetum	(Meigen,1818)	
Polypedilum (Polypedilum) nubeculosum	(Meigen,1804)	*
Chironomus (Chironomus) riparius	Meigen,1804	*
Chironomus (Chironomus) crassimanus	Strenzke,1959	
Stictochironomus pictulus	(Meigen,1830)	
Paracladopelma camptolabis	(Kieffer,1913)	*

Table S3. Pearson correlation coefficients of correlations between environmental variables and taxa abundances. Significant values are in bold in colored cells (**p<0.01). Abbreviations of variable and species names are provided in Tables 1 and 2.

	altit	dist	depth	velcorr	flowtype	temp	cond	alcal	pH	NO3	NH4	O2	sed	As	Cd	Cu	Hg	sostorg	DDT	Ni	Pb
**altit**		**-0.95**	**0.25**	0.19	-0.10	-0.01	0.21	**0.37**	**0.69**	**0.49**	0.06	-0.08	-0.09	**-0.66**	-0.22	0.12	**-0.58**	-0.09	**-0.43**	**0.56**	**-0.40**
**dist**	**		**-0.30**	-0.16	0.01	0.04	-0.18	**-0.31**	**-0.63**	**-0.42**	-0.08	0.09	0.17	**0.59**	0.17	-0.03	**0.50**	-0.02	**0.40**	**-0.54**	**0.40**
**depth**	**	**		0.08	0.09	0.02	**0.35**	0.14	0.07	0.17	0.16	0.15	-0.06	-0.21	0.01	0.07	**-0.29**	-0.17	0.09	0.09	-0.07
**velcorr**					**0.64**	0.09	0.16	0.01	0.12	-0.06	0.01	0.14	0.02	-0.22	0.06	0.15	-0.13	-0.07	-0.10	0.08	0.06
**flowtype**				**		-0.01	-0.11	0.02	0.04	-0.15	0.18	-0.02	-0.22	0.08	**0.28**	0.14	0.17	0.22	0.17	-0.12	0.11
**temp**							0.06	-0.15	**0.41**	-0.15	-0.20	**0.51**	**0.30**	**0.27**	0.18	**0.38**	**-0.37**	**-0.34**	**0.31**	-0.15	**0.27**
**cond**			**					**0.25**	-0.08	**0.44**	0.17	**0.33**	-0.22	-0.20	-0.21	-0.07	-0.10	-0.20	-0.07	0.17	**-0.31**
**alcal**	**	**					**		0.20	**0.53**	**0.80**	-0.05	-0.21	**-0.37**	0.23	0.09	-0.12	0.14	0.05	**0.29**	-0.04
**pH**	**	**				**				0.08	-0.04	0.05	-0.09	-0.23	-0.11	0.20	**-0.59**	-0.05	-0.20	**0.26**	-0.24
**NO3**	**	**					**	**			0.23	-0.05	0.12	**-0.35**	**-0.32**	-0.19	**-0.36**	**-0.32**	-0.06	0.16	**-0.40**
**NH4**								**				-0.15	**-0.31**	**-0.27**	**0.46**	-0.01	0.14	**0.27**	**0.26**	0.05	0.01
**O2**						**	**						0.09	0.16	-0.10	0.02	-0.17	-0.15	0.00	-0.03	0.12
**sed**						**					**			0.05	-0.06	0.04	-0.12	**-0.39**	0.17	-0.17	**0.26**
**As**	**	**				**		**		**	**				-0.03	**-0.27**	0.17	0.06	**0.31**	**-0.53**	0.14
**Cd**					**					**	**					**0.56**	**0.36**	**0.29**	**0.40**	-0.12	**0.75**
**Cu**						**								**	**		0.01	-0.04	0.21	**0.30**	**0.55**
**Hg**	**	**	**			**			**	**					**			**0.61**	0.05	-0.02	**0.25**
**sostorg**						**				**	**		**		**		**		**-0.41**	0.19	0.16
**DDT**	**	**				**					**			**	**			**		-0.23	**0.27**
**Ni**	**	**						**	**		0.05			**		**					-0.12
**Pb**	**	**				**	**			**			**		**	**	**		**		
**Matro**	-0.21	0.22	-0.23	-0.03	0.03	**-0.39**	-0.14	0.04	**-0.27**	-0.08	0.07	-0.14	-0.01	0.08	-0.04	-0.11	**0.38**	**0.29**	-0.09	0.06	0.00
**Microt**	0.21	-0.21	-0.01	-0.05	-0.11	0.23	**-0.32**	-0.14	**0.29**	-0.18	-0.19	-0.10	0.17	-0.12	0.19	**0.27**	-0.10	0.06	-0.08	0.20	**0.28**
**Polyp**	-0.14	0.16	-0.23	-0.06	-0.14	-0.16	-0.19	-0.08	-0.21	-0.09	-0.09	-0.11	0.10	-0.07	-0.04	0.06	0.19	0.16	-0.18	0.13	0.18
**Tvet**	-0.09	0.03	0.11	0.17	**0.31**	**-0.27**	0.07	-0.09	-0.15	-0.10	-0.03	0.08	-0.14	0.09	-0.09	-0.25	**0.28**	**0.31**	-0.16	0.08	-0.11
**Odont**	-0.18	0.19	-0.04	-0.18	-0.19	**-0.42**	-0.24	0.05	**-0.35**	-0.09	0.13	**-0.32**	0.05	-0.11	**0.27**	0.09	**0.34**	0.18	0.00	0.02	**0.28**
**Mpelop**	-0.13	0.10	-0.08	-0.02	0.02	0.05	-0.01	0.05	-0.17	0.04	0.06	0.17	0.01	-0.01	0.12	0.07	0.06	0.07	0.04	0.03	0.24
**Symp**	-0.02	-0.01	**-0.37**	-0.20	-0.21	-0.15	0.11	0.00	-0.08	0.11	-0.06	0.07	-0.15	0.13	**-0.33**	**-0.26**	0.17	**0.33**	**-0.32**	0.21	-0.24
**Diam**	0.03	-0.08	0.12	0.12	0.07	-0.14	0.24	-0.02	-0.03	0.05	-0.03	0.12	-0.14	0.07	**-0.32**	**-0.32**	0.11	0.23	**-0.27**	0.15	**-0.30**
**Concha**	0.02	-0.02	0.20	0.06	-0.01	-0.11	0.03	0.11	-0.14	0.03	0.19	-0.06	-0.05	-0.16	0.12	0.07	-0.07	-0.03	0.05	0.03	0.08
**Prod**	-0.09	0.12	-0.14	-0.09	-0.17	-0.09	-0.14	0.08	-0.15	-0.09	0.12	-0.14	0.13	-0.10	**0.27**	0.14	0.23	0.11	0.03	0.05	0.25
**Euk**	0.05	-0.04	-0.13	-0.01	0.06	**0.25**	-0.21	**-0.28**	**0.28**	-0.12	-0.22	0.01	0.10	0.03	0.01	0.11	-0.09	-0.09	0.11	-0.10	0.03
**Pclad**	-0.02	0.07	-0.13	0.03	-0.06	**-0.25**	-0.15	-0.05	-0.09	-0.08	-0.07	-0.16	0.08	-0.11	-0.10	0.02	0.01	-0.04	-0.12	0.00	-0.01

**Monod**	-0.16	0.21	-0.01	0.01	-0.07	0.16	-0.03	-0.01	-0.09	-0.15	0.06	0.05	0.17	0.04	0.13	0.16	0.03	-0.09	0.13	-0.11	0.13
**Orth**	0.01	0.09	-0.19	0.05	-0.19	**-0.29**	-0.01	0.11	-0.10	0.03	0.08	-0.15	0.09	-0.12	-0.12	0.02	0.09	0.02	-0.17	0.09	-0.08
**Ctrem**	0.09	-0.10	0.01	0.04	0.07	-0.16	0.04	0.05	-0.01	0.17	0.05	-0.13	-0.14	-0.09	-0.16	-0.15	-0.07	-0.08	-0.01	0.00	**-0.28**
**Cbici**	-0.10	0.18	-0.06	-0.01	-0.11	-0.08	-0.02	0.00	-0.15	-0.05	0.00	-0.06	0.13	-0.02	0.05	0.12	0.11	-0.04	-0.02	-0.07	0.09
**Rheoc**	-0.18	0.25	-0.20	-0.06	-0.15	**-0.25**	0.04	-0.09	**-0.26**	-0.07	-0.06	-0.16	0.07	0.02	-0.09	-0.01	**0.31**	0.07	-0.09	-0.02	-0.07
**Euorth**	0.10	-0.12	0.05	0.09	0.09	-0.25	0.01	0.05	0.09	-0.06	0.05	0.01	-0.16	0.01	**-0.31**	**-0.30**	0.05	**0.30**	**-0.31**	0.20	**-0.29**
**Cripa**	-0.05	0.00	**0.30**	-0.17	-0.15	0.13	0.18	-0.02	-0.13	-0.05	0.16	0.02	-0.05	-0.04	0.11	0.07	-0.02	-0.09	0.21	0.02	0.02
**Synor**	-0.13	0.18	-0.06	-0.04	-0.14	-0.07	-0.01	-0.06	-0.17	0.07	-0.12	-0.04	0.23	0.05	-0.07	0.00	0.05	-0.15	0.07	-0.07	0.09
**Cardio**	-0.11	0.14	0.01	0.16	0.08	0.06	0.14	0.00	-0.09	0.15	-0.09	0.10	0.19	0.16	-0.10	-0.13	-0.08	-0.25	**0.27**	-0.03	0.06
**Ptrich**	-0.15	0.10	-0.12	-0.10	-0.03	-0.09	-0.01	-0.08	-0.11	-0.10	-0.06	0.03	-0.12	0.16	-0.08	-0.09	0.24	**0.32**	-0.13	0.06	-0.02
**Potth**	0.04	-0.07	0.18	-0.05	0.01	-0.07	0.03	0.17	-0.05	0.11	**0.25**	-0.13	-0.10	-0.09	0.07	0.00	-0.09	-0.04	0.18	0.00	-0.06
**Chaet**	-0.04	0.06	0.07	-0.03	-0.08	-0.11	0.16	-0.12	-0.16	0.05	-0.11	-0.06	-0.02	-0.04	-0.14	-0.09	0.14	-0.05	-0.10	-0.02	-0.17
**Thimmyia**	-0.06	0.03	-0.23	-0.14	-0.11	-0.13	-0.04	-0.03	-0.05	-0.12	-0.02	0.08	-0.13	0.10	-0.14	-0.12	0.15	**0.30**	-0.18	0.11	-0.07
**Cory**	-0.02	0.03	0.01	-0.04	-0.05	0.06	0.05	0.04	0.00	0.00	0.04	**0.36**	0.01	0.01	-0.10	-0.08	-0.08	-0.07	0.01	0.05	-0.08
**Metr**	-0.06	0.03	-0.06	-0.05	0.16	-0.10	-0.10	0.17	-0.08	0.03	0.20	-0.09	-0.08	0.00	0.19	-0.05	0.15	0.09	0.08	-0.08	0.07
**Bril**	0.13	-0.13	-0.04	0.04	0.03	0.05	0.02	0.10	0.17	0.09	0.06	-0.03	0.00	-0.05	-0.11	-0.08	-0.06	0.08	-0.13	0.18	-0.12
**Ofrig**	0.08	-0.08	-0.06	0.09	0.20	0.14	-0.15	-0.15	0.18	-0.05	-0.16	-0.01	0.04	0.00	0.04	0.16	-0.06	-0.03	0.02	-0.03	0.04
**Psectr**	-0.11	0.11	0.06	-0.03	-0.02	0.12	0.02	0.00	-0.04	-0.04	0.01	**0.41**	0.05	0.07	0.00	0.00	-0.01	-0.06	0.09	-0.01	0.03
**Tany**	-0.14	0.19	-0.06	-0.08	-0.10	0.11	0.07	0.01	0.00	-0.05	0.01	**0.32**	-0.01	0.08	-0.02	-0.03	-0.03	-0.14	0.19	-0.02	0.04
**Ptriss**	0.09	-0.09	-0.09	-0.07	-0.12	-0.14	0.01	0.01	0.03	-0.03	0.01	0.02	-0.03	-0.08	-0.17	-0.14	-0.06	0.02	-0.14	0.07	-0.15

Table S4. CCA results, inertia and eigenvalues of the first canonical axes according to: **a**) full CCA, **b**) partial CCA.

	a) CCA with all variables			b) partial CCA			
	Total	Constrained	Unconstrained	Total	Conditioned	Constrained	Unconstrained
**Inertia**	8.063	2.558	5.506	8.064	0.298	0.420	7.346
**Proportion**	1.000	0.317	0.683	1.000	0.037	0.052	0.911
	**Eigenvalue**	**Proportion Explained**	**Cumulative Proportion**		**Eigenvalue**	**Proportion Explained**	**Cumulative Proportion**
**CCA1**	0.502	0.062	0.062	**CCA1**	0.257	0.033	0.033
**CCA2**	0.379	0.047	0.109	**CCA2**	0.094	0.012	0.045
**CCA3**	0.289	0.036	0.145	**CCA3**	0.068	0.009	0.054
**CCA4**	0.240	0.030	0.175	**CA1**	0.577	0.074	0.128
**CCA5**	0.209	0.026	0.201	**CA2**	0.534	0.069	0.197
**CCA6**	0.190	0.024	0.224	**CA3**	0.520	0.067	0.264
**CCA7**	0.152	0.019	0.243	**CA4**	0.472	0.061	0.325

Table S5. Results of CCA with all environmental variables. Scores of environmental variables according to CCA axes. Abbreviations of variable names are provided in Table 1.

	**CCA1**	**CCA2**	**CCA3**	**CCA4**	**CCA5**	**CCA6**
**pH**	0.183	-0.360	0.135	0.010	0.060	-0.025
**temp**	0.314	-0.259	0.309	-0.077	-0.051	-0.025
**O2**	-0.131	-0.229	0.340	0.022	-0.213	0.077
**As**	-0.152	-0.182	0.049	0.022	-0.049	0.002
**flowtype**	-0.024	-0.116	0.123	0.265	0.077	0.020
**Ni**	-0.176	-0.115	-0.024	-0.074	0.052	0.005
**sostorg**	-0.234	-0.075	-0.129	-0.039	0.024	0.006
**velcorr**	-0.012	-0.057	0.083	0.208	0.011	-0.087
**NO3**	-0.135	-0.001	0.037	0.018	0.144	0.068
**cond**	-0.314	0.005	0.286	0.007	0.109	-0.101
**sed**	0.241	0.055	-0.097	0.005	-0.185	-0.070
**Hg**	-0.166	0.116	-0.206	0.015	-0.063	-0.059
**Cu**	0.427	0.152	-0.068	-0.094	-0.119	-0.012
**DDT**	0.297	0.165	0.138	0.073	-0.020	0.147
**Pb**	0.322	0.179	-0.158	0.001	-0.169	0.087
**alcal**	-0.113	0.216	0.000	-0.042	-0.012	0.132
**depth**	0.087	0.221	0.309	0.205	0.129	-0.049
**Cd**	0.346	0.274	-0.077	0.030	-0.085	0.085
**NH4**	-0.056	0.314	0.073	-0.044	0.025	0.149

Table S6. Results of CCA: scores of species according to CCA axes for: a) CCA considering all environmental variables; b) partial CCA. Abbreviations of species names are provided in Table 3.

	**a) CCA with all variables**						**b) partial CCA**					
	**CCA1**	**CCA2**	**CCA3**	**CCA4**	**CCA5**	**CCA6**	**CCA1**	**CCA2**	**CCA3**	**CA1**	**CA2**	**CA3**
Matro	-0.559	0.076	-0.635	0.386	-0.290	0.352	0.035	0.037	0.313	0.079	-0.035	0.168
Microt	1.772	-0.883	-0.009	0.004	-0.038	-0.861	0.022	-0.136	-0.430	-1.003	0.393	-0.306
Polyp	0.083	-0.227	-0.698	-0.630	0.024	-0.080	0.149	-0.198	-0.194	-0.101	-0.091	0.068
Tvet	-0.754	-0.142	0.607	1.923	-0.128	-0.425	0.266	0.068	0.184	0.252	0.250	0.854
Odont	0.315	1.154	-1.020	0.453	0.018	0.549	-0.464	-0.188	0.097	-0.510	-0.419	-0.103
Mpelop	0.029	-0.143	0.610	-0.039	-0.399	1.474	-0.155	-0.070	0.010	-0.150	-0.361	0.634
Symp	-1.401	-1.184	-0.252	-1.433	0.369	0.321	0.857	0.132	0.001	0.427	-0.045	0.186
Diam	-1.176	-0.360	0.960	0.848	0.571	-1.223	0.606	0.198	-0.091	0.637	0.066	0.051
Concha	0.293	1.482	1.168	-0.144	0.920	0.972	-0.703	0.312	-0.333	0.371	-0.235	-0.089
Prod	0.735	0.836	-0.801	-0.591	-0.846	-0.344	-0.414	-0.803	-0.047	-0.379	-0.374	-0.236
Euk	2.155	-2.114	0.302	0.302	0.445	0.338	-0.170	0.137	-0.001	-1.175	1.775	-0.700
Pclad	0.256	0.204	-0.713	-0.377	-0.546	0.046	-0.182	0.413	-0.337	-0.387	-0.059	-0.203
Monod	1.096	0.855	1.618	-1.572	-1.142	-1.403	-0.274	-0.099	0.190	0.889	-2.005	-2.298
Orth	-0.698	0.391	-0.922	-0.744	-0.959	-0.462	-0.049	0.102	-0.386	-0.054	0.054	0.152
Ctrem	-0.194	0.185	0.465	0.119	2.459	0.910	-0.533	0.655	-0.268	0.776	0.586	0.366
Cbici	0.292	1.440	-0.244	-0.989	-1.956	-2.174	-0.129	-0.318	0.064	-0.196	-1.371	-0.779
Rheoc	-0.620	0.364	-0.739	-0.695	-0.449	-1.657	0.132	-0.384	0.247	0.064	0.292	-0.121
Euorth	-1.657	-0.558	0.439	0.450	0.162	-0.660	0.853	0.487	-0.339	0.350	0.301	0.150
Cripa	0.824	1.951	3.678	-1.748	1.587	-0.448	-0.966	-0.059	0.141	2.038	0.058	-1.771
Synor	0.183	-0.053	-0.975	1.007	-0.363	0.074	-0.380	0.468	0.426	-0.176	0.119	0.134
Cardio	-0.225	-1.157	-0.096	4.196	0.572	1.988	-0.937	1.447	1.091	-0.369	-1.105	1.865
Ptrich	-1.176	-1.160	-0.452	-1.037	0.896	-0.160	1.330	-0.340	0.746	0.393	0.099	-0.037
Potth	0.561	2.217	1.973	-0.992	3.433	2.917	-1.596	0.796	0.007	4.576	3.310	-0.628
Chaet	-0.943	0.829	0.571	0.757	1.675	-4.253	0.290	-0.657	-0.225	0.543	0.317	0.450
Thimmyia	-1.420	-1.335	-0.240	-2.490	-0.676	1.589	1.078	0.086	0.233	0.181	0.037	-0.174
Cory	-1.030	-0.416	3.220	-0.188	-4.255	2.169	-0.096	0.556	-0.115	0.652	-1.030	3.180
Metr	-0.009	1.403	-0.222	1.616	-0.545	0.901	-0.706	-0.351	0.529	0.283	0.302	1.833
Bril	-0.629	-1.400	0.340	-0.546	0.615	-1.494	0.573	-0.026	-0.766	0.217	0.057	0.471
Ofrig	3.014	-3.101	0.149	1.400	0.211	-0.223	0.008	0.023	-0.214	-2.329	2.958	-1.089
Psectr	-0.622	-0.344	4.949	1.487	-7.231	2.830	-0.162	0.271	0.677	0.694	-1.224	4.188
Tany	0.393	-1.661	2.320	1.403	-4.091	3.608	-0.840	0.643	0.869	0.069	-0.317	1.004
Ptriss	-1.733	-0.736	0.648	-3.129	-2.401	1.862	0.314	0.939	-1.145	-0.019	0.082	-0.289

Table S7. Partial CCA: scores of constraining variables of the first three CCA axes.

	**CCA1**	**CCA2**	**CCA3**
**As**	0.435	0.205	0.862
**Hg**	0.156	-0.661	0.617
**DDT**	-0.743	0.016	0.575

Table S8. Results of SOM: quantization and topographic error, and percent of total variance explained by the clustering. Abbreviations of variable names are provided in Table 1.

	Quantization error	Percent variance explained	Topographic error
**dist**	7.03	77.8	0.766
**depth**	6.75	78.7	0.664
**month**	4.43	86.0	0.505
**temp**	7.46	76.5	0.645
**habitat**	4.99	84.3	0.589
**microh**	8.70	72.5	0.682
**flowtype**	6.17	80.6	0.664
**vel**	8.55	73.0	0.589
**cond**	7.93	75.0	0.636
**pH**	8.03	74.7	0.692
**O2**	9.56	69.8	0.542
**orgmat**	7.58	76.1	0.561
**Cd**	6.72	78.8	0.645
**Cu**	7.31	76.9	0.664
**Ni**	5.76	81.8	0.607
**Pb**	7.54	76.2	0.542
**As**	7.46	76.5	0.692
**Hg**	6.46	79.6	0.636
**DDT**	6.88	78.3	0.617

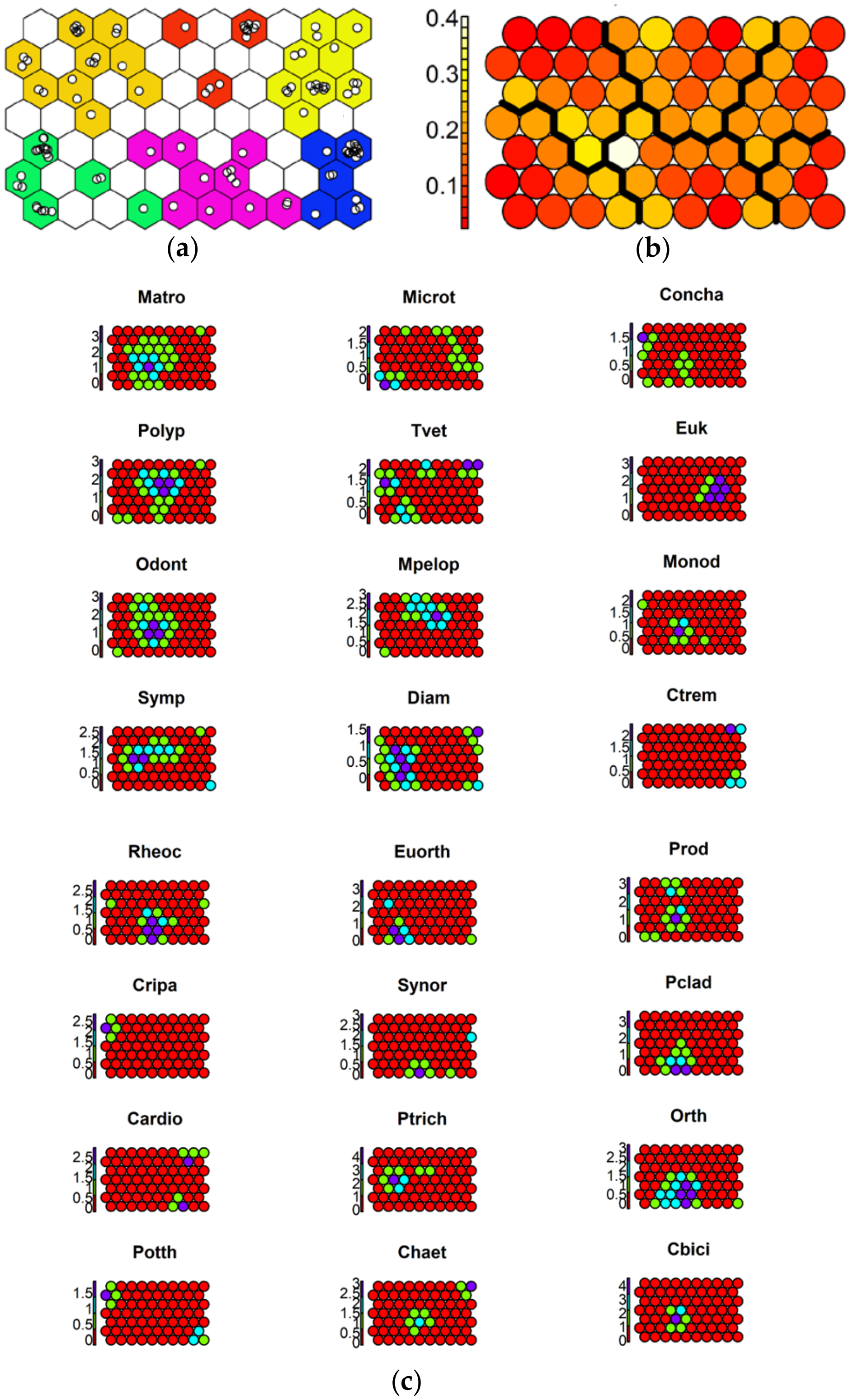

Figure S1: Results of SSOM analysis using distance from the source as an ordering external factor: **a**) sites mapped in 6 clusters, with different colors: 49-52 km (blue cells), 52-58 km (green cells), 58-63 km (yellow cells), 63-67 km (orange cells), 67-72 km (red cells); **b**) distances between clusters: high distances in yellow, low distances in red; **c**) species graphs representing the different abundances of a species in the cells (values on y axis represent log_{10}-tranformed abundance): very abundant (purple cells), abundant (cyan cells), present (green cells), absent (red cells). Abbreviations of species names are reported in Table 3.

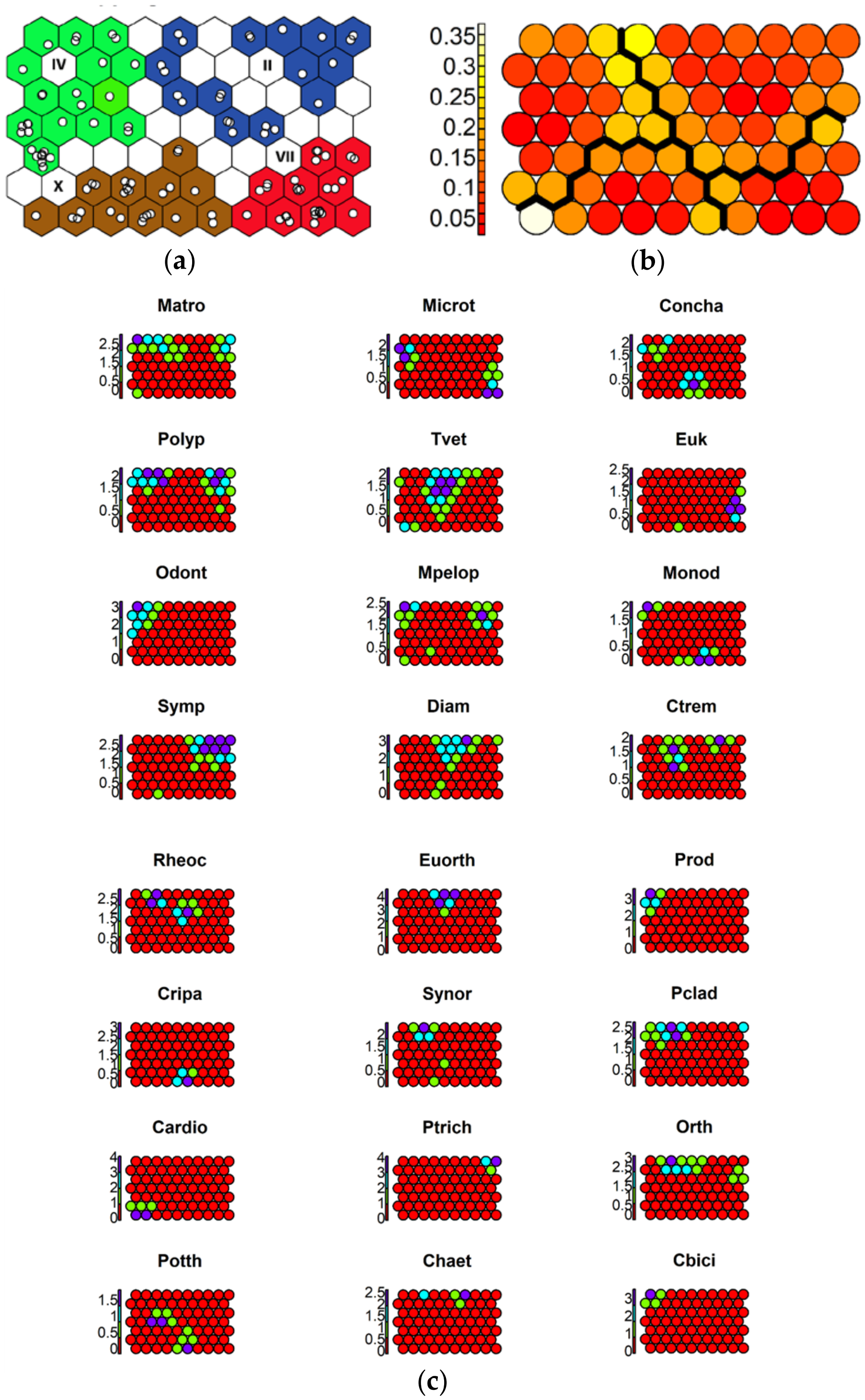

Figure S2. Results of SSOM analysis using sampling month as an ordering external factor: **a**) sites mapped in 4 clusters with different colors: February (II), April (IV), July (VII) and October (X); **b**) distances between clusters: high distances in yellow, low distances in red; **c**) species graphs representing the different abundances of a species in the cells (values on y axis represent log_{10}-tranformed abundance): very abundant (purple cells), abundant (cyan cells), present (green cells), absent (red cells). Abbreviations of species names are reported in Table 3.

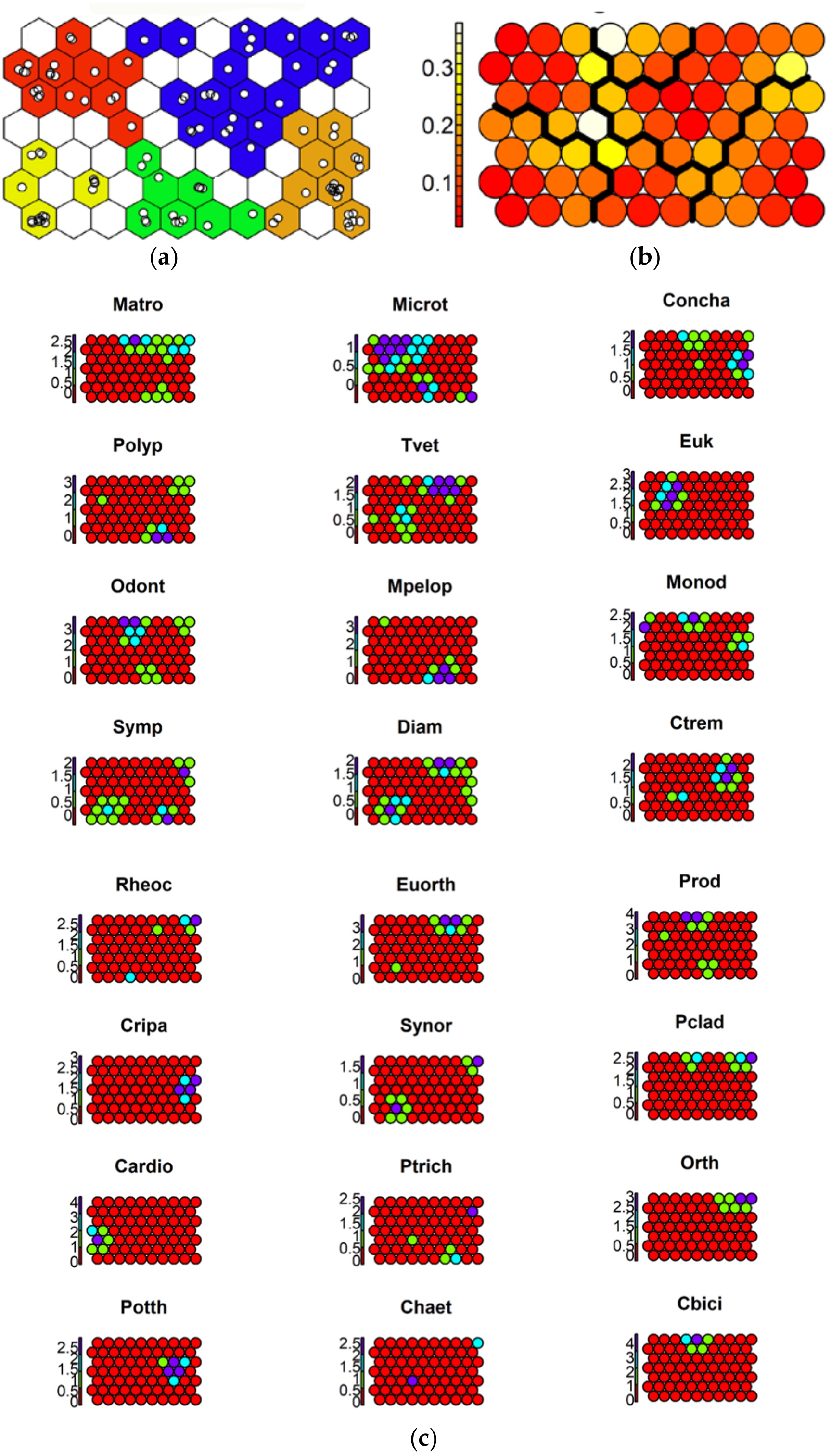

Figure S3. Results of SSOM analysis using water temperature as an ordering external factor: a) sites mapped in 5 clusters, with different colors: <8 °C (blue cells), 8-10 °C (green cells), 10-12 °C (yellow cells), 12-16 °C (orange cells), >16 °C (red cells); b) distances between clusters: high distances in yellow, low distances in red; c) species graphs representing the different abundances of a species in the cells (values on y axis represent $\log_{10}$-tranformed abundance): very abundant (purple cells), abundant (cyan cells), present (green cells), ab-sent (red cells). Abbreviations of species names are reported in Table 3.

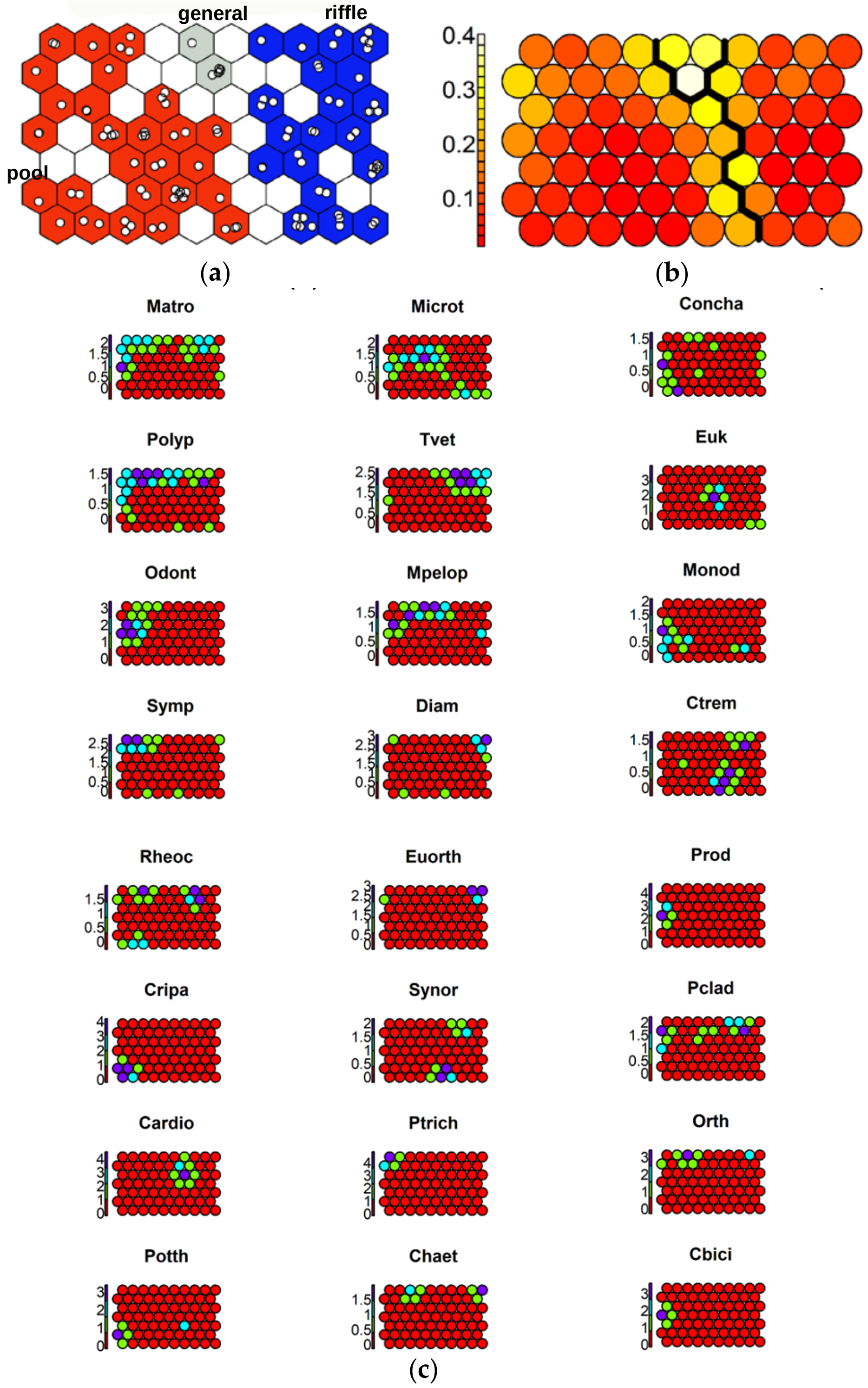

Figure S4: Results of SSOM analysis using mesohabitat as an ordering external factor: **a**) sites mapped in 3 clusters, with different colors: riffles (blue cells), pools (red cells), general survey (grey cells) (i.e., where pools and riffles could not be clearly separated); **b**) distances between clusters: high distances in yellow, low distances in red; **c**) species graphs representing the different abundances of a species in the cells (values on y axis represent $\log_{10}$-tranformed abundance): very abundant (purple cells), abundant (cyan cells), present (green cells), absent (red cells). Abbreviations of species names are reported in Table 3.

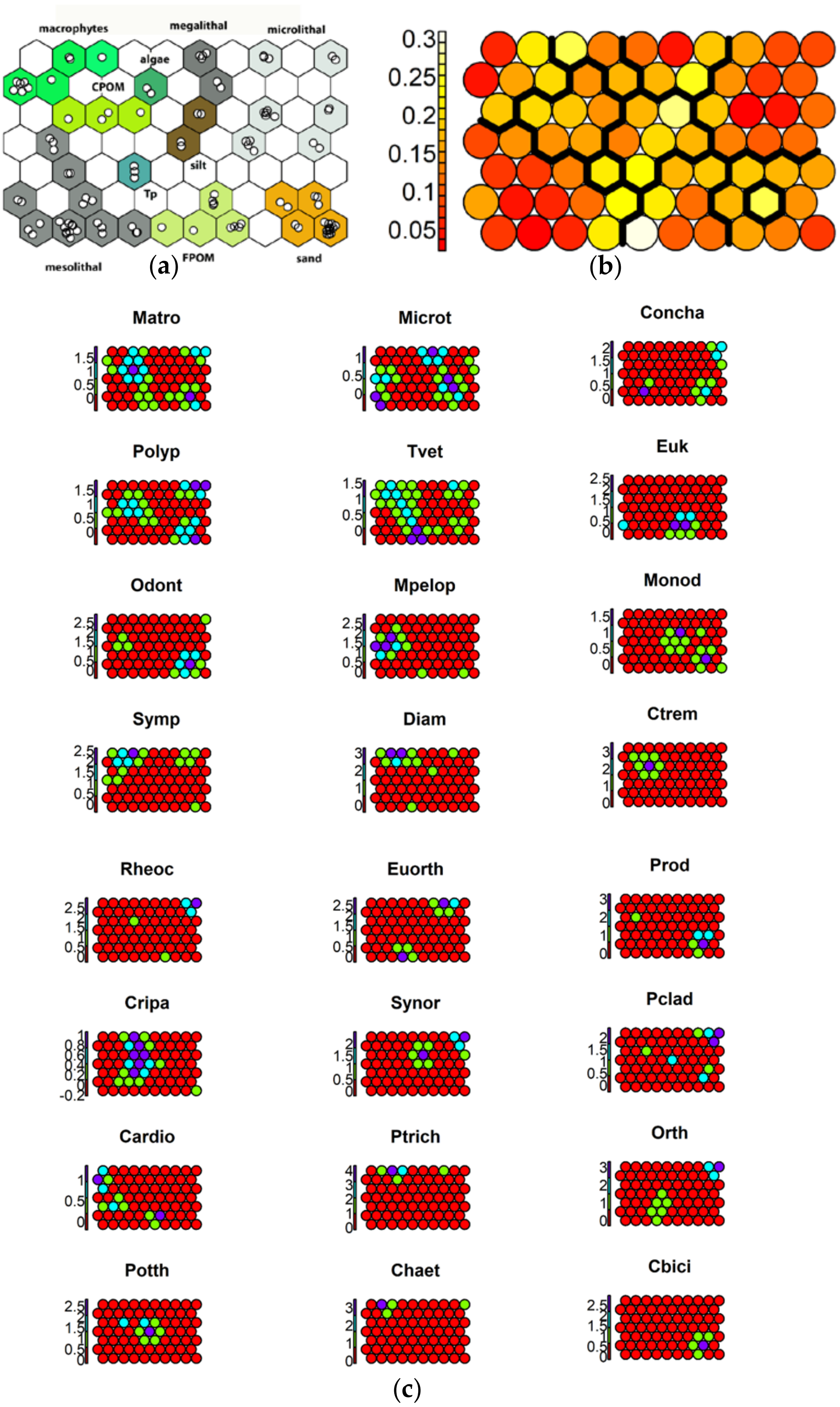

Figure S5. Results of SSOM analysis using microhabitat as an ordering external factor: a) sites mapped in 10 clusters, with different colors: microlithal, mesolithal, megalithal, sand, CPOM, FPOM, algae, macrophytes, silt, Tp; b) distances between clusters: high distances in yellow, low distances in red; c) species graphs representing the different abundances of a species in the cells (values on y axis represent $\log_{10}$-tranformed abundance): very abundant (purple cells), abundant

(cyan cells), present (green cells), absent (red cells). Abbreviations of species names are reported in Table 3.

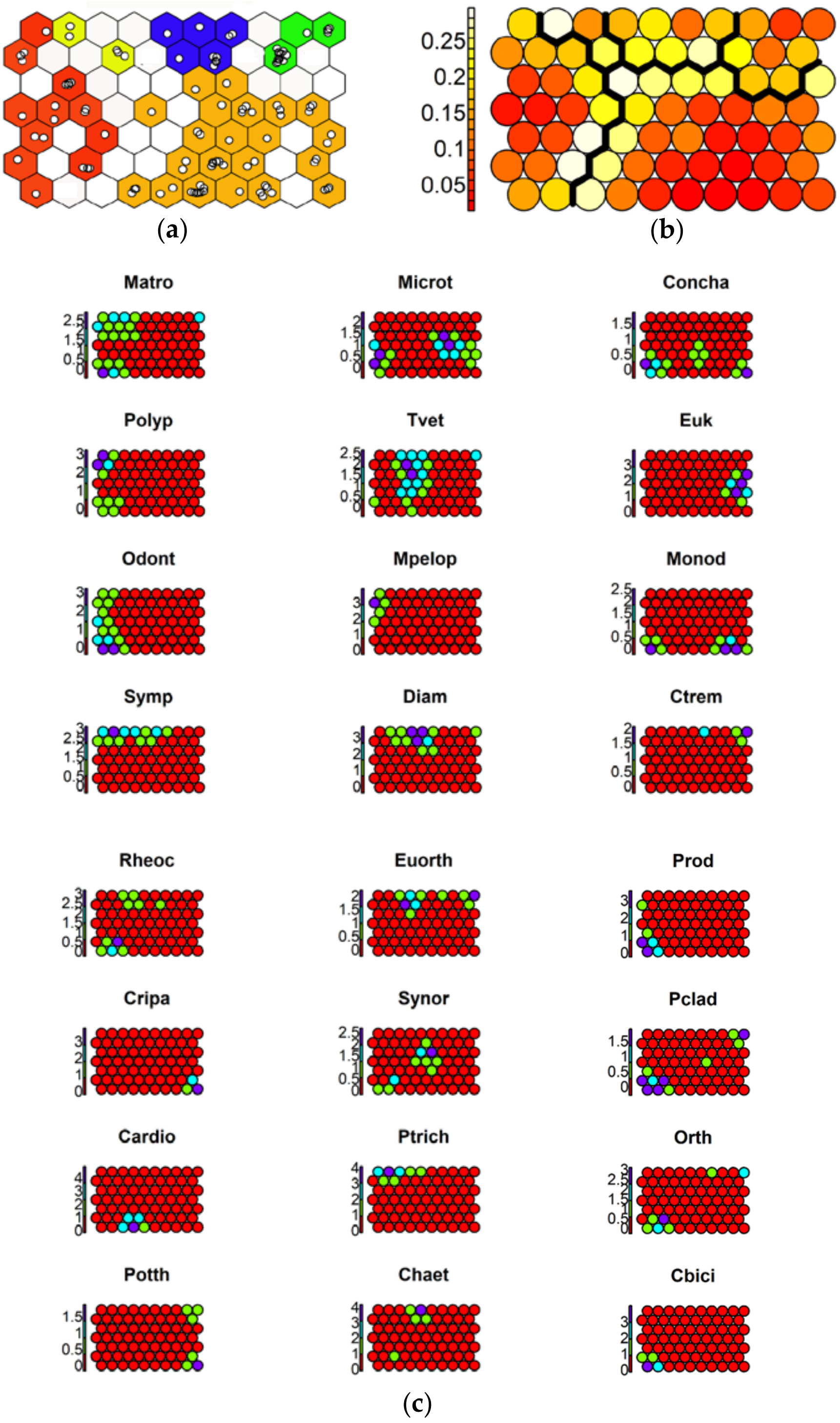

Figure S6. Results of SSOM analysis using Pb as an ordering external factor: a) sites mapped in 5 clusters, with different colors: <9 mg kg^{-1} d.w. (blue cells), 9-10 mg kg^{-1} d.w. (green cells), 10-12 mg kg^{-1} d.w. (yellow cells), 12-15 mg kg^{-1} d.w. (orange cells), >15 mg kg^{-1} d.w. (red cells); b) distances between clusters: high distances in yellow, low distances in red; c) species graphs representing the different abundances of a species in the cells (values on y axis represent log$_{10}$-tranformed

abundance): very abundant (purple cells), abundant (cyan cells), present (green cells), absent (red cells). Abbreviations of species names are reported in Table 3.

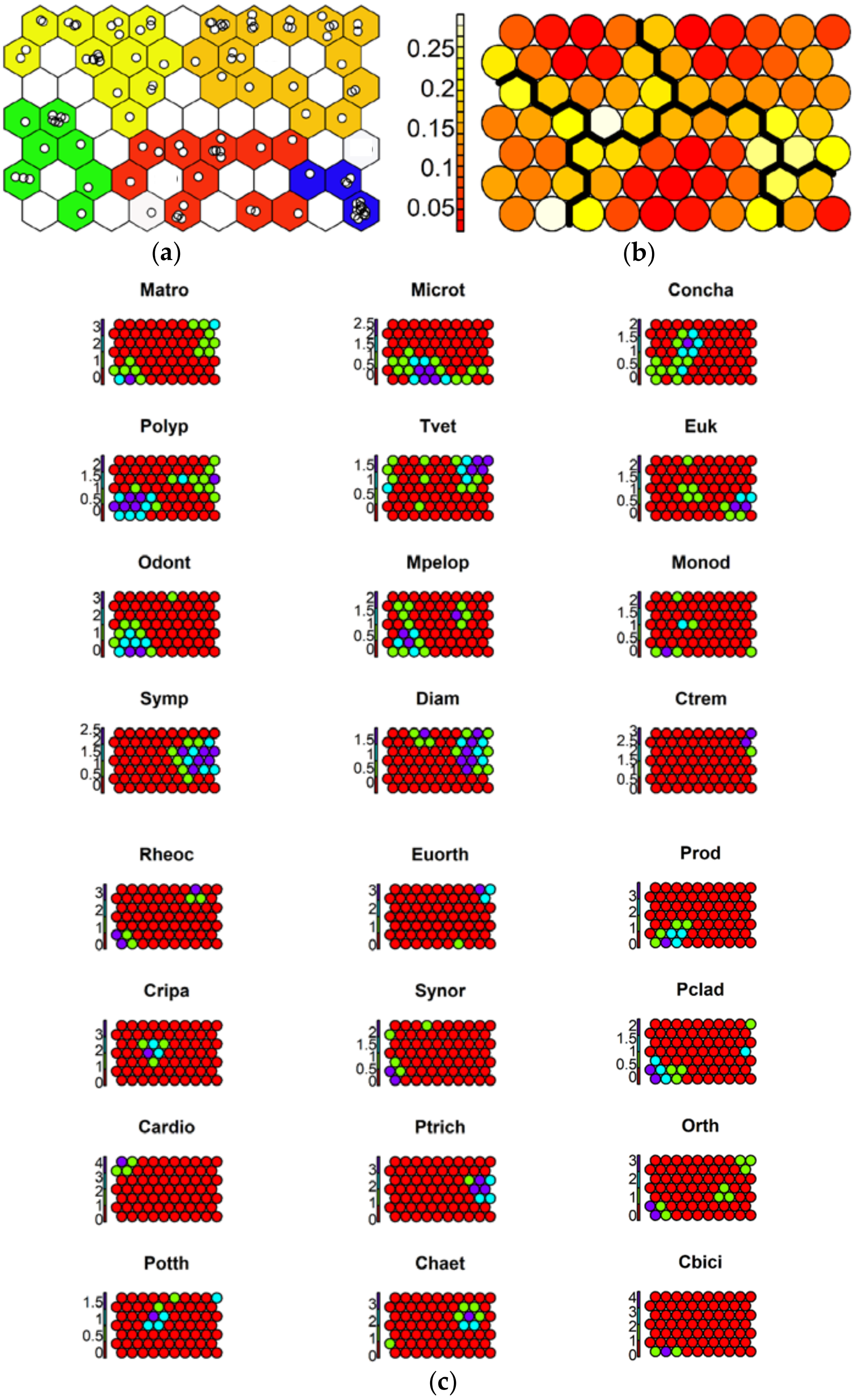

Figure S7. Results of SSOM analysis using Ni as an ordering external factor: a) sites mapped in 5 clusters, with different colors: <20 mg kg^{-1} d.w. (blue cells), 20-25 mg kg^{-1} d.w. (green cells), 25-30 mg kg^{-1} d.w. (yellow cells), 30-40 mg kg^{-1} d.w. (orange cells), >40 mg kg^{-1} d.w. (red cells); b) distances between clusters: high distances in yellow, low distances in red; c) species graphs representing the different abundances of a species in the cells (values on y axis represent log_{10}-tranformed

abundance): very abundant (purple cells), abundant (cyan cells), present (green cells), absent (red cells). Abbreviations of species names are reported in Table 3.

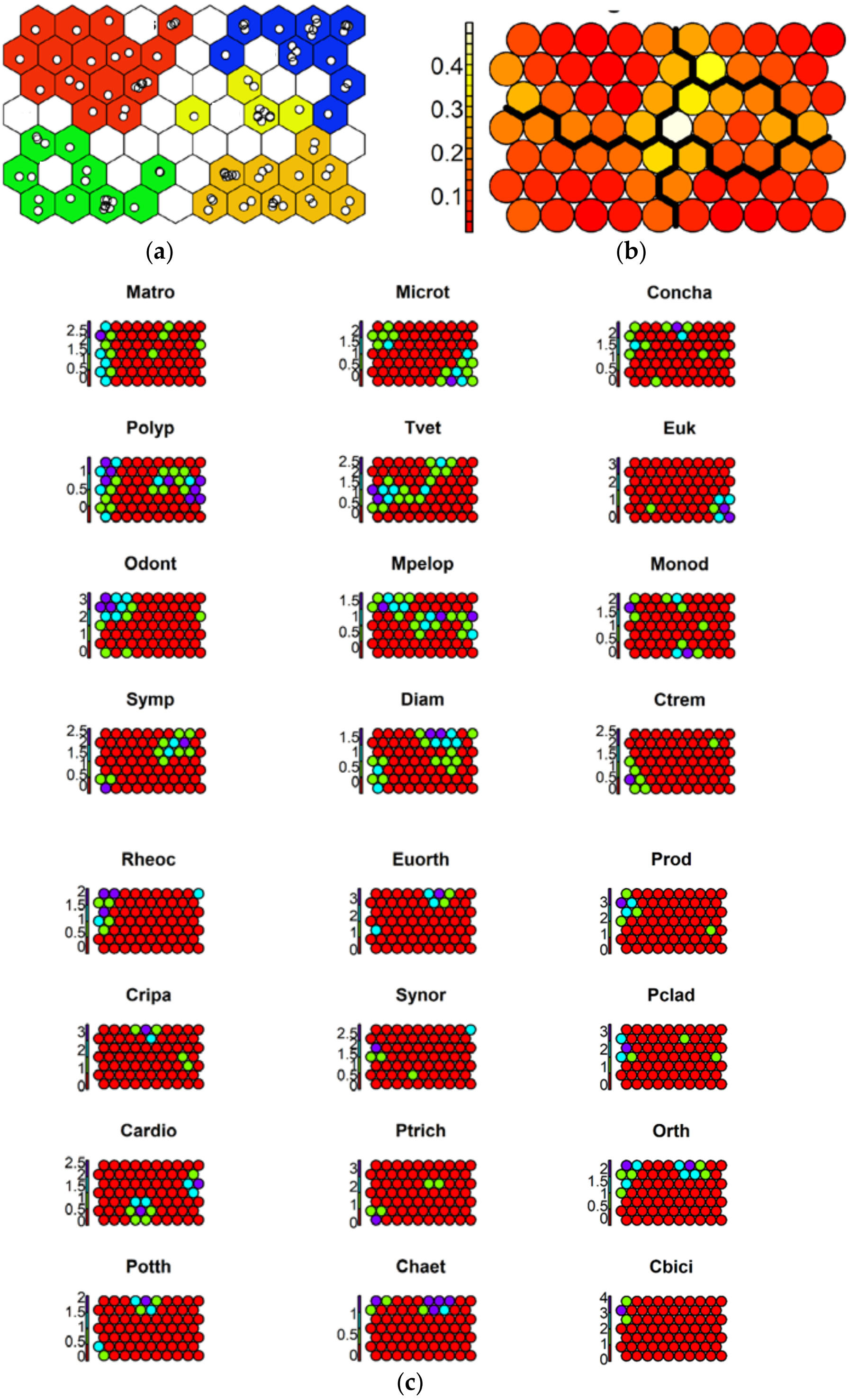

Figure S8. Results of SSOM analysis using Cd as an ordering external factor: a) sites mapped in 5 clusters, with different colors: : <125 μg kg^{-1} d.w. (blue cells), 125-140 μg kg^{-1} d.w. (green cells), 140-160 μg kg^{-1} d.w. (yellow cells), 160-175 μg kg^{-1} d.w. (orange cells), >175 μg kg^{-1} d.w. (red cells); b) distances between clusters: high distances in yellow, low distances in red; c) species graphs representing the different abundances of a species in the cells (values on y axis represent log$_{10}$-

tranformed abundance): very abundant (purple cells), abundant (cyan cells), present (green cells), absent (red cells). Abbreviations of species names are reported in Table 3.

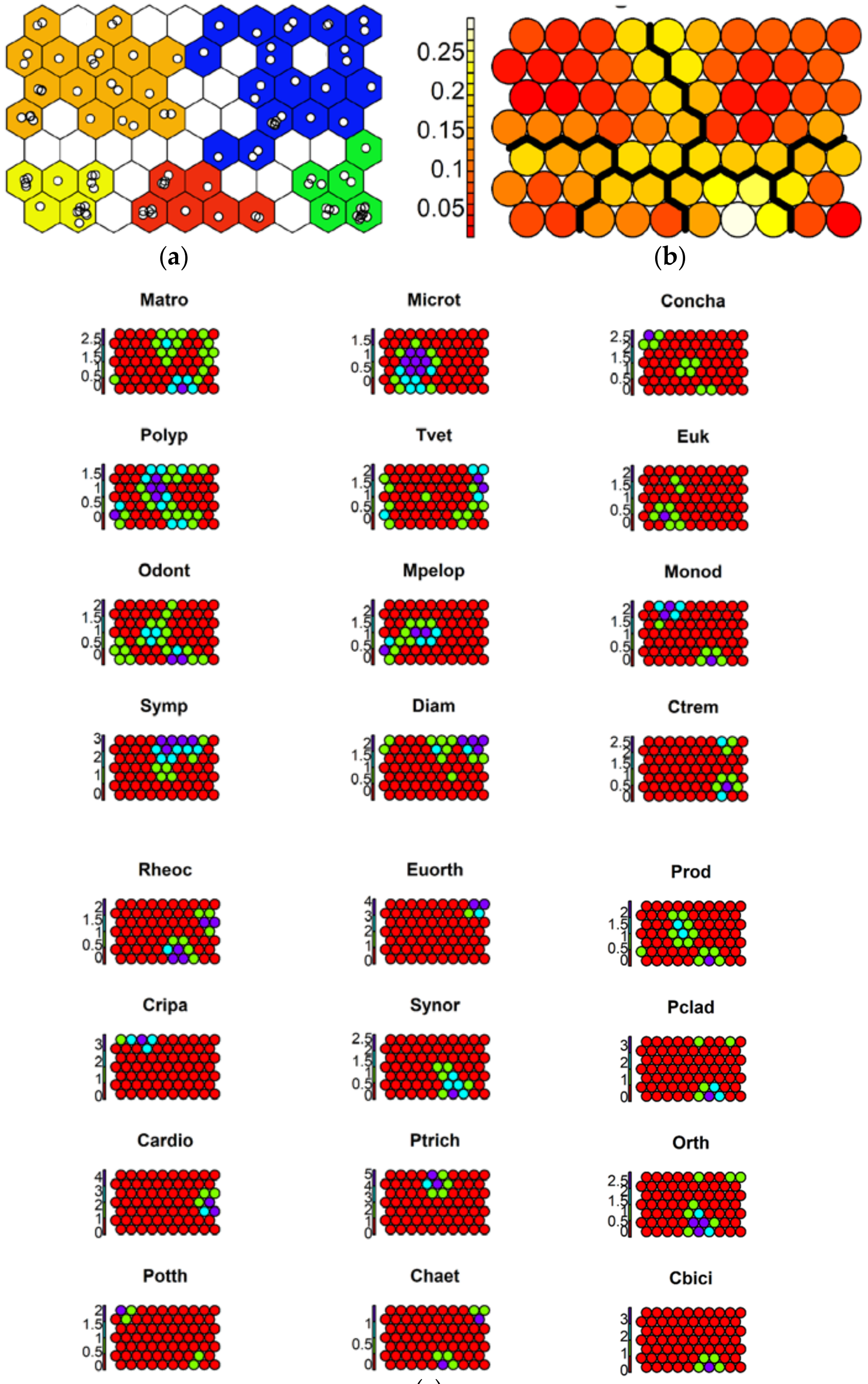

Figure S9. Results of SSOM analysis using Cu as an ordering external factor: a) sites mapped in 5 clusters, with different colors: : <20 mg kg^{-1} d.w. (blue cells), 20-25 mg kg^{-1} d.w. (green cells), 25-30 mg kg^{-1} d.w. (yellow cells), 30-40 mg kg^{-1} d.w. (orange cells), >40 mg kg^{-1} d.w. (red cells); b) distances between clusters: high distances in yellow, low distances in red; c) species graphs representing the different abundances of a species in the cells (values on y axis represent log_{10}-tranformed

abundance): very abundant (purple cells), abundant (cyan cells), present (green cells), absent (red cells). Abbreviations of species names are reported in Table 3.

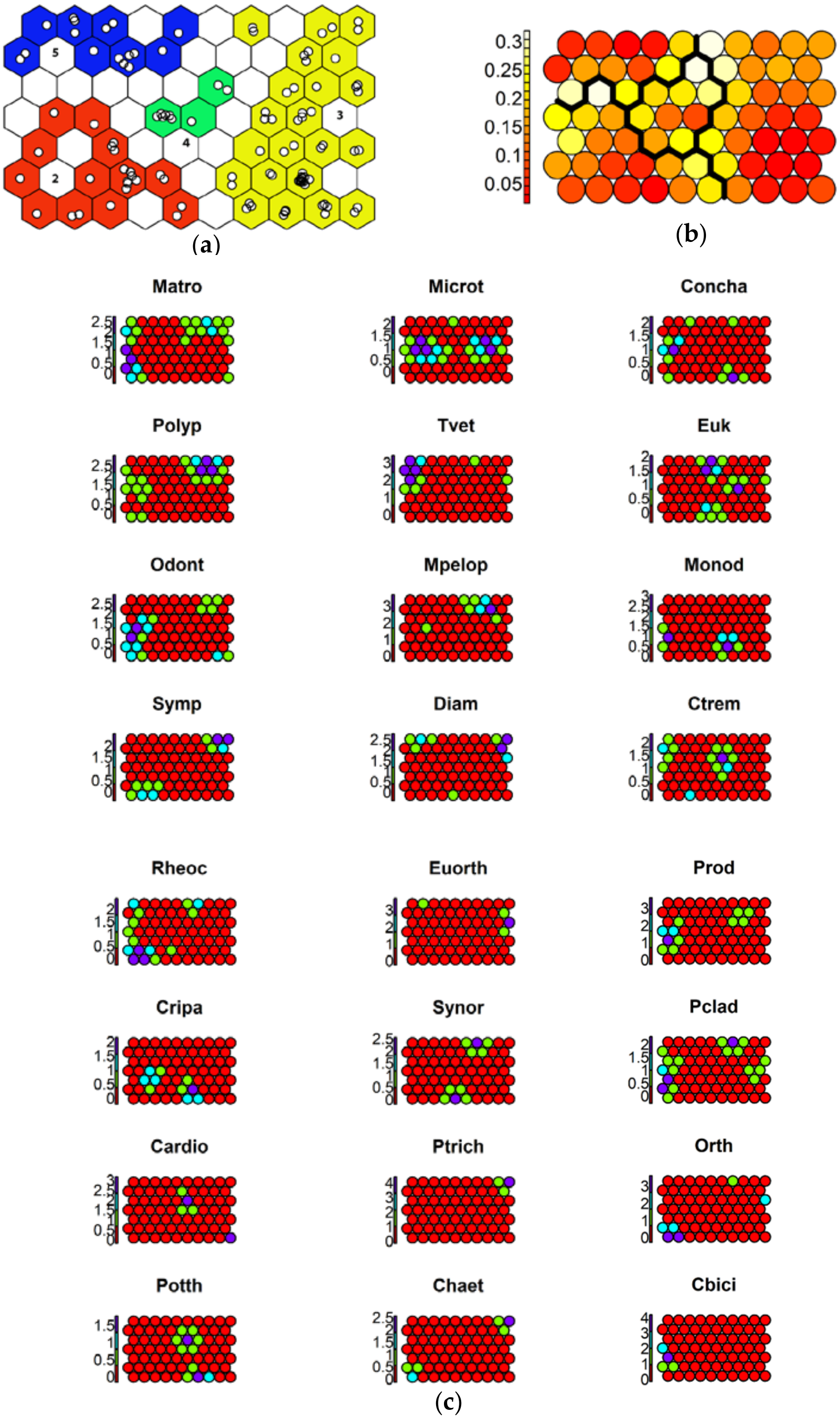

Figure S10. Results of SSOM analysis using flow type as an ordering external factor: a) sites mapped in 4 clusters, with different colors: rippled (2), unbroken standing waves (3), broken standing waves (4), chute (5); b) distances between clusters: high distances in yellow, low distances in red; c) species graphs representing the different abundances of a species in the cells (values on y axis represent

log10-tranformed abundance): very abundant (purple cells), abundant (cyan cells), present (green cells), absent (red cells). Abbreviations of species names are reported in Table 3.

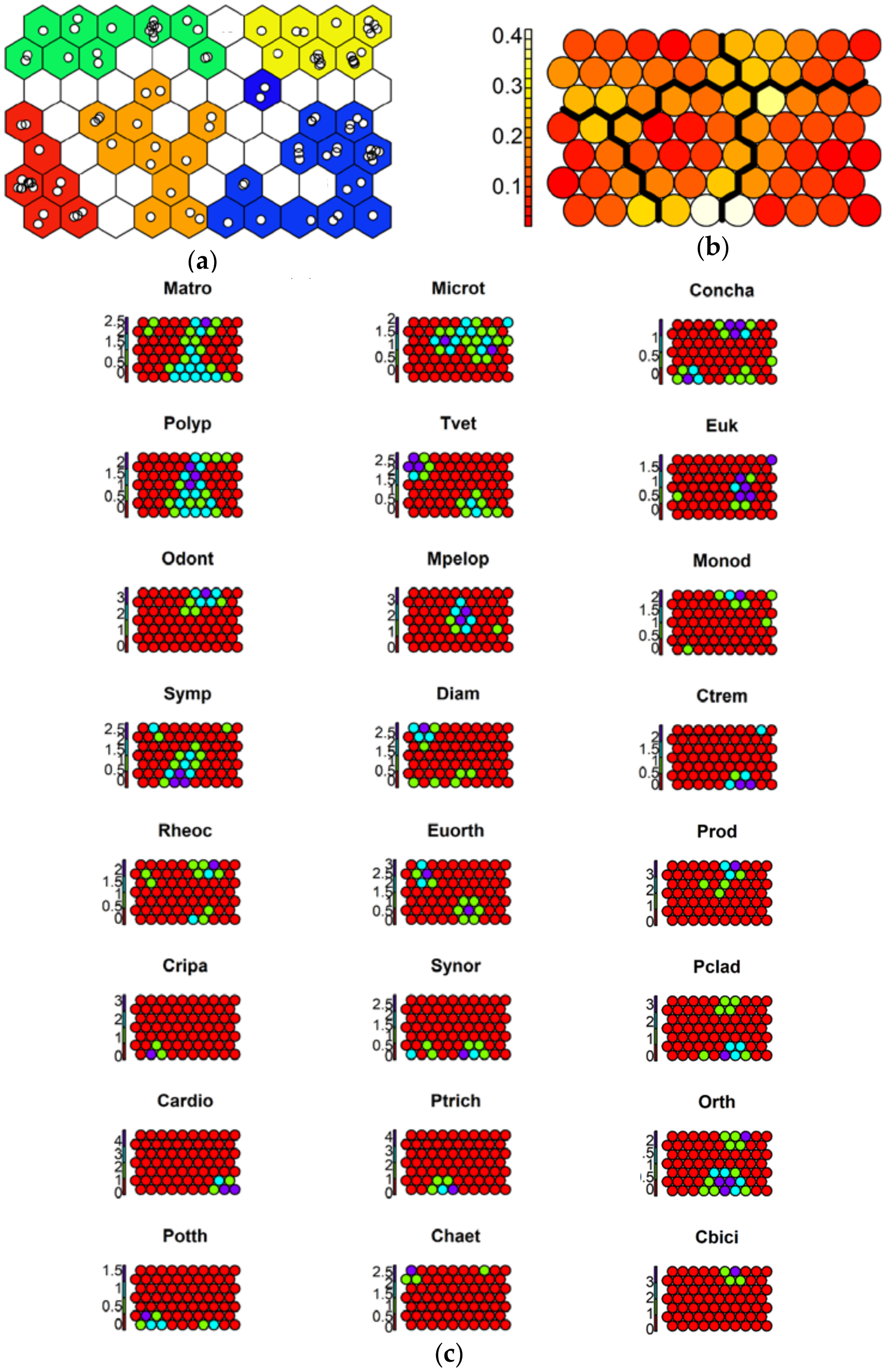

Figure S11. Results of SSOM analysis using current velocity as an ordering external factor: a) sites mapped in 5 clusters, with different colors: : >25 cm s^{-1} (blue cells), 15-25 cm s^{-1} (green cells), 10-15 cm s^{-1} (yellow cells), 5-10 cm s^{-1} (orange cells), <5 cm s^{-1} (red cells); b) distances between clusters: high distances in yellow, low distances in red; c) species graphs representing the different abundances of

a species in the cells (values on y axis represent log_{10}-tranformed abundance): very abundant (purple cells), abundant (cyan cells), present (green cells), absent (red cells). Abbreviations of species names are reported in Table 3.

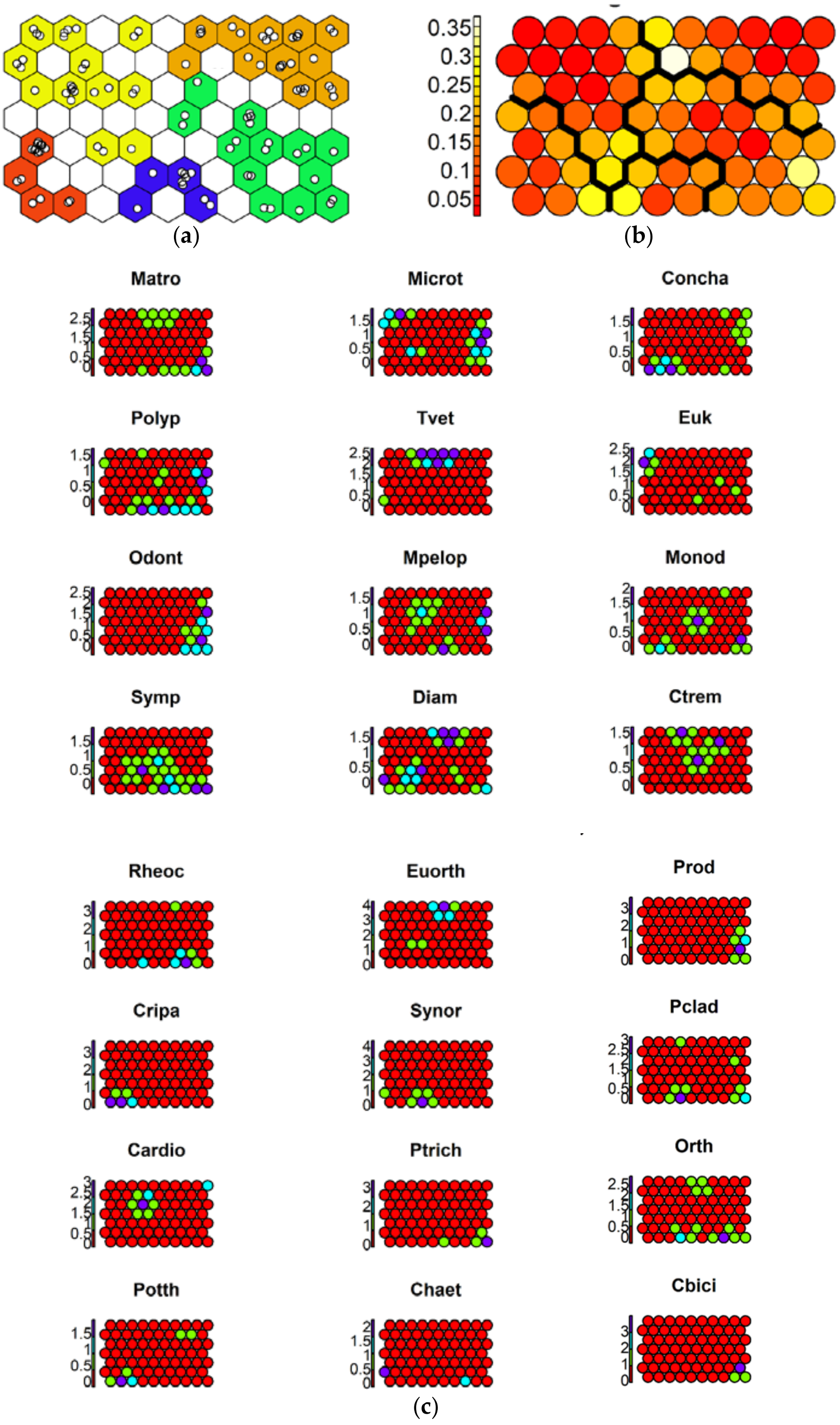

Figure S12. Results of SSOM analysis using water depth as an ordering external factor: a) sites mapped in 5 clusters, with different colors: : <15 cm (blue cells), 15-20 cm (green cells), 20-25 cm (yellow cells), 25-30 cm (orange cells), >30 cm (red cells); b) distances between clusters: high distances

in yellow, low distances in red; c) species graphs representing the different abundances of a species in the cells (values on y axis represent log_{10}-tranformed abundance): very abundant (purple cells), abundant (cyan cells), present (green cells), absent (red cells). Abbreviations of species names are reported in Table 3.

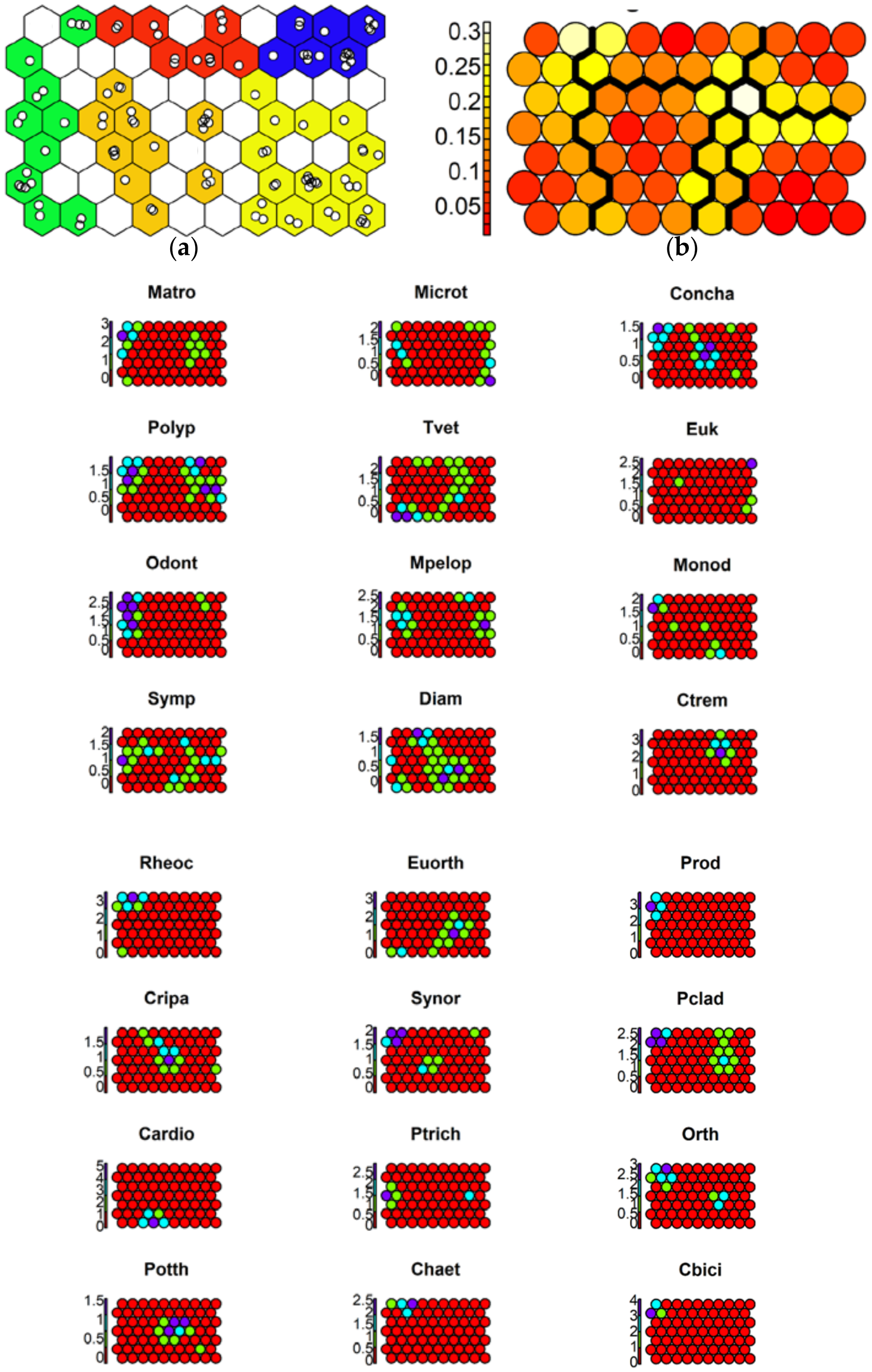

(c)

Figure S13. Results of SSOM analysis using water conductivity as an ordering external factor: a) sites mapped in 5 clusters, with different colors: <150 μS cm^{-1} (blue cells), 150-160 μS cm^{-1} (green

cells), 160-170 µS cm^{-1} (yellow cells), 170-220 µS cm^{-1} (orange cells), >220 µS cm^{-1} (red cells); b) distances between clusters: high distances in yellow, low distances in red; c) species graphs representing the different abundances of a species in the cells (values on y axis represent log$_{10}$-tranformed abundance): very abundant (purple cells), abundant (cyan cells), present (green cells), absent (red cells). Abbreviations of species names are reported in Table 3.

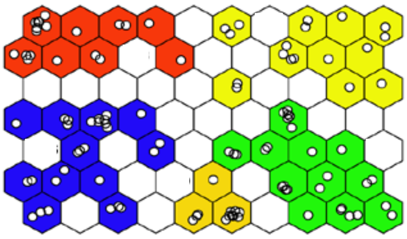

(a)

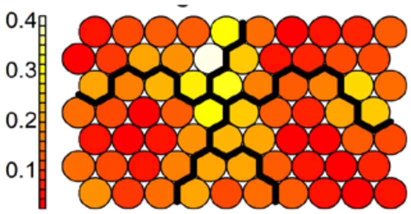

(b)

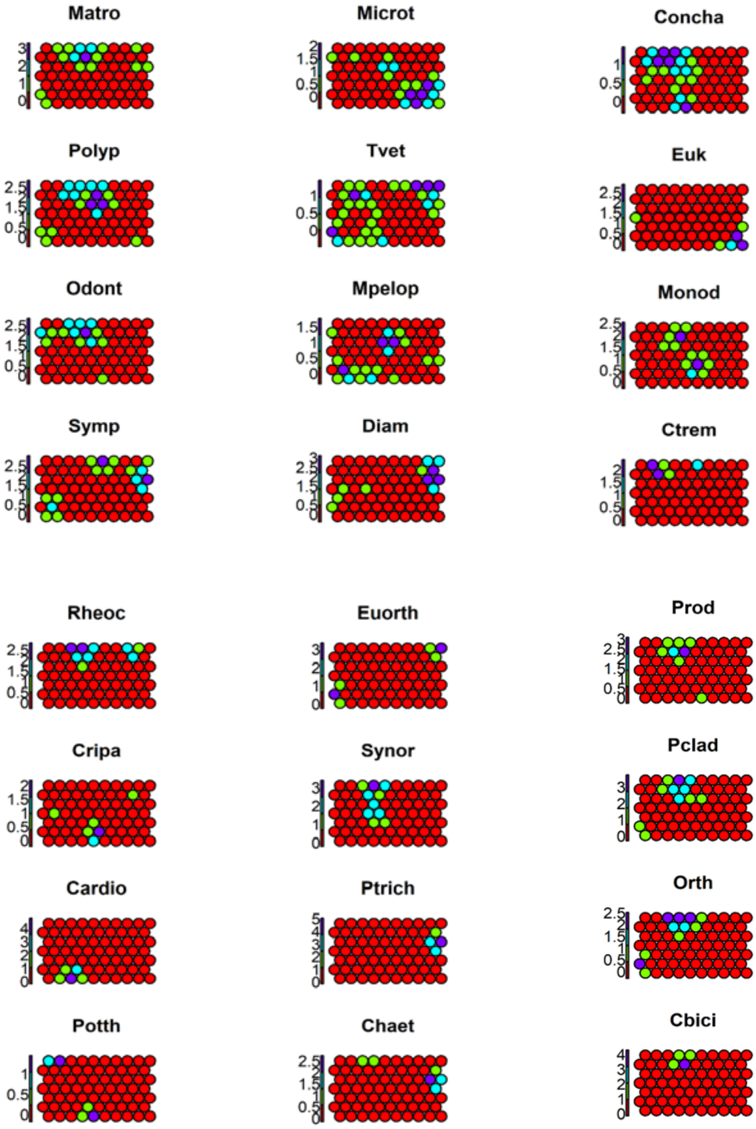

(c)

Figure S14. Results of SSOM analysis using oxygen saturation as an ordering external factor: a) sites mapped in 5 clusters, with different colors: >105% (blue cells), 102-105% (green cells), 101-102% (yellow cells), 100-101% (orange cells), <98 % (red cells); b) distances between clusters: high distances in yellow, low distances in red; c) species graphs representing the different abundances of a species in the cells (values on y axis represent log_{10}-tranformed abundance): very abundant (purple cells), abundant (cyan cells), present (green cells), absent (red cells). Abbreviations of species names are reported in Table 3.

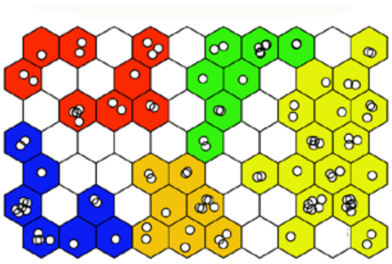

(**a**)

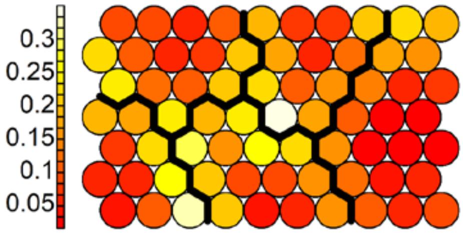

(**b**)

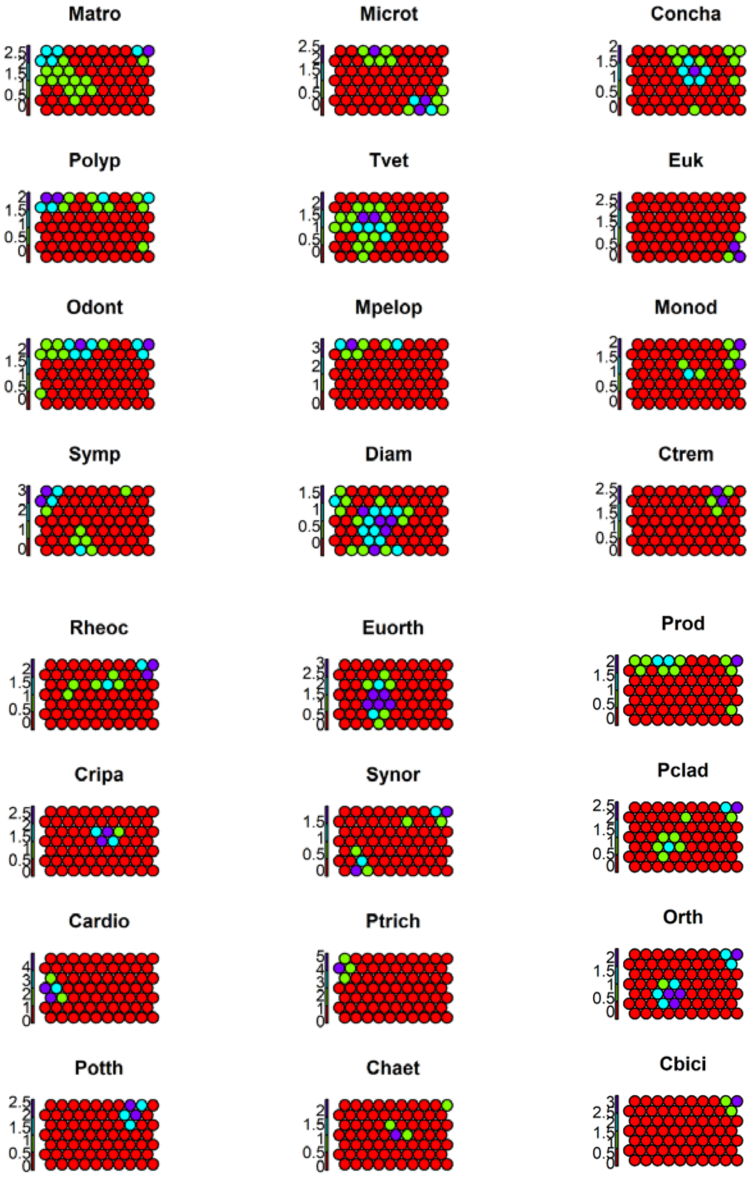

(**c**)

Figure S15. Results of SSOM analysis using organic carbon in sediments as an ordering external factor: a) sites mapped in 5 clusters, with different colors: <1 % (blue cells), 1.0-1.2 % (green cells), 1.2-1.5 % (yellow cells), 1.5-3.2 % (orange cells), >3.2 % (red cells); b) distances between clusters: high distances in yellow, low distances in red; c) species graphs representing the different abundances of a species in the cells (values on y axis represent log_{10}-tranformed abundance): very abundant (purple cells), abundant (cyan cells), present (green cells), absent (red cells). Abbreviations of species names are reported in Table 3.
